# Socioeconomic status and long-term risk of inflammatory bowel disease: a prospective cohort study with mediation analysis

**DOI:** 10.7189/jogh.16.04391

**Published:** 2026-11-06

**Authors:** Qian Zhang, Yutao Wang, Shuang Yu, Yesheng Zhou, Si Liu, Peng Li, Shutian Zhang, Shanshan Wu

**Affiliations:** 1 Department of Gastroenterology, Beijing Friendship Hospital, Capital Medical University, Beijing, China; 2 State Key Laboratory of Digestive Health, Beijing, China; 3 National Clinical Research Center for Digestive Diseases, Beijing, China; 4 Beijing Key Laboratory of Early Gastrointestinal Cancer Medicine and Medical Devices, Beijing, China

**Keywords:** socioeconomic status, healthy lifestyle, inflammatory bowel disease, cohort study

## Abstract

**Background:**

The association of socioeconomic status with incident inflammatory bowel disease and the contribution of lifestyle factors remain unclear. We aimed to comprehensively investigate the association between socioeconomic status (SES) and the risk of inflammatory bowel disease (IBD), and to assess the mediating effect of lifestyle factors in a prospective cohort.

**Methods:**

We assessed SES using household income, education level, and employment status, and categorised participants into high-, medium-, and low-SES groups using latent profile analysis. We combined lifestyle factors, including smoking, alcohol consumption, diet, physical activity, body mass index, and sleep duration, to calculate a healthy lifestyle score as a mediator. The primary outcome was incident IBD. We used Cox proportional hazards models and mediation analysis to examine associations between SES and incident IBD and to assess the mediation effect.

**Results:**

Participants free of IBD at baseline were included (n = 374,507), with a mean age of 55.84 years, of whom 51.5% were female. Overall, 76,292 (20.4%) were classified as high, 197,130 (52.6%) as medium, and 101,085 (27.0%) as low patterns. Over 14.57 years of follow-up, we identified 2381 incident IBD cases, 1703 ulcerative colitis (UC) and 843 Crohn's disease (CD), with an overall incidence rate of 44.9 per 100,000 person-years. After multivariable adjustment, a high SES pattern was associated with a 45% lower IBD risk compared to low SES (hazard ratio (HR) = 0.55; 95% confidence interval (CI) = 0.48–0.63). Meanwhile, all SES components showed beneficial effects, with higher education level demonstrating the strongest association (HR = 0.58; 95% CI = 0.51–0.65). The combination of high SES and healthy lifestyle conferred the greatest protection (HR = 0.37; 95% CI = 0.29–0.46). Mediation analyses indicated that lifestyle factors explained 11.34–16.50% of the observed association between SES and incident IBD. Similar protective associations were observed for both UC and CD.

**Conclusions:**

Healthy lifestyle factors partially explained the association between SES and incident IBD. However, these findings should be interpreted cautiously given the observational design, potential residual confounding, and assumption-dependent mediation estimates.

Inflammatory bowel disease (IBD), including Crohn's disease (CD) and ulcerative colitis (UC), is a chronic inflammatory disorder affecting the gastrointestinal tract. As a major public health challenge, IBD significantly impairs quality of life and imposes a substantial financial burden on the healthcare system [1]. Thus, understanding the multifaceted aetiology of IBD is crucial for developing effective prevention and treatment strategies. Recent studies have suggested that various environmental, genetic, and lifestyle factors significantly contribute to the pathogenesis of IBD, yet the underlying mechanisms remain poorly elucidated [2].

Socioeconomic status (SES), an individual's position in society that systematically challenges health equity, is an important determinant of health outcomes [3]. Low SES has been demonstrated to be associated with higher risks of mortality and other adverse health outcomes [4]. Conceptual frameworks linking SES to chronic inflammatory diseases propose that social inequality translates into biological damage through five interconnected pathways: stress-induced immune dysregulation, hazardous environmental exposures, inadequate healthcare access, poor dietary quality, and gut microbiome disruption. Several cross-sectional studies reported a higher prevalence of IBD in high-income areas such as Europe, North America, and Oceania than in low-income areas. A 21% higher IBD incidence in urban areas has also been reported [5–8]. Conversely, previous case-control or cohort studies reported no association between income, education level, and IBD risk [9,10], while another population-based study in Canada found unemployment rates among IBD patients were approximately 2-fold higher than in the general population [9]. These inconsistencies may reflect several methodological limitations, including reliance on cross-sectional designs, relatively small sample sizes, use of single SES indicators that fail to capture the multidimensional nature of socioeconomic position, and inadequate control of key confounding factors [11]. Additionally, SES may shape lifestyle behaviours through differences in health literacy and access to healthy food environments and recreational spaces, while lifestyle factors, including smoking, diet quality, physical activity, body mass index (BMI), and sleep duration, have been independently associated with IBD risk. Therefore, we hypothesised that healthy lifestyle factors could mediate the association between SES and incident IBD [12–16]. However, the mediating role of lifestyle factors in the relationship between SES and risk of incident IBD, along with the joint effects of SES and lifestyle factors on IBD risk, has not yet been thoroughly examined.

Therefore, in this large-scale prospective cohort study, we aimed to investigate the effect of SES on incident IBD risk, assess the mediating effects of lifestyle factors, and evaluate the joint effects of SES and lifestyle factors on IBD risk.

## METHODS

We conducted and reported this secondary analysis in accordance with the Journal of Global Health’s GRABDROP [17] and STROBE guidelines [18] (Tables S1 and S2 in the **Online Supplementary Document**).

### Study population

The UK Biobank (UKB) is an ongoing large-scale prospective cohort study. Between 2006 and 2010, researchers recruited over half a million participants aged 37–73 years across 22 centres in England, Wales, and Scotland. At baseline, all participants completed questionnaires, underwent anthropometric assessments, and provided medical history information. Further details regarding UKB cohort design have been described elsewhere [19].

Participants free of IBD with available SES and lifestyle data at enrolment were included. For this analysis, we excluded individuals with prevalent IBD at baseline (n = 5,159), prevalent cancer (n = 46,629), missing baseline data (n = 71,839) and missing lifestyle factors (n = 4,182), resulting in a sample size of 374,507 participants included (Figure S1 in the **Online Supplementary Document**).

### SES assessment

We assessed SES at baseline *via* a questionnaire using three indicators, household income, education level, and employment status, selected to capture multiple socioeconomic dimensions (material resources, cognitive capacity, and occupational roles), consistent with the measurement approach used in previous large-scale studies [15]. The available options for household income included ‘<GBP 18,000’, ‘GBP 18,000–30,999’, ‘GBP 31,000–51,999’, ‘GBP 52,000–100,000’ and ‘>GBP 100,000’. Educational qualifications were recorded as ‘college or university degree’, ‘Advanced levels/Advanced Subsidiary levels or equivalent’, ‘Ordinary levels/General Certificates of Secondary Education or equivalent’, ‘Certificates of Secondary Education or equivalent’, ‘National Vocational Qualifications or Higher National Diploma or Higher National Certificate or equivalent’, ‘other professional qualifications’ and ‘none of the above’ (equivalent to less than a high school diploma). Employment status was categorised as employed (including ‘in paid employment or self-employed’, ‘retired’, ‘doing unpaid or voluntary work’, and ‘full- or part-time students’) and unemployed (including ‘looking after home and/or family’, ‘unable to work because of sickness or disability’, and ‘unemployed’). Consequently, we identified overall SES patterns (low, medium, and high) based on these three components using LCA, a model-based probabilistic clustering approach designed to detect latent groupings [20]. We determined the optimal number of latent classes by evaluating model fit statistics, including the Akaike Information Criterion, Bayesian Information Criterion, and likelihood ratio χ^2^ statistic. We also used the mean posterior probability, which reflects uncertainty in posterior classification, for model selection, with values of 0.7 or higher indicating acceptable uncertainty (Methods S1, Table S3, and Figure S2 in the **Online Supplementary Document**).

We also calculated SES scores for each participant. We categorised household income into three groups: high (≥GBP 52,000; score = 2), medium (GBP 18,000–51,999; score = 1), and low (<GBP 18,000; score = 0). Education level was classified as college or higher (score = 2), high school or equivalent (including Advanced/Advanced Subsidiary levels, Ordinary levels/General Certificates of Secondary Education, Certificates of Secondary Education, National Vocational Qualifications or Higher National Diploma or Higher National Certificate, and other professional qualifications; score = 1), and less than high school (none of the above; score = 0). Employment status was scored as 0 for unemployed and 2 for employed. We summed SES scores (ranging from 0 to 6), with higher scores indicating greater socioeconomic status. We further grouped participants into three SES categories: 0–2 (reference group), 3–4, and 5–6.

### Assessment of healthy lifestyle score

We collected lifestyle behaviours at recruitment *via* a self-administered touch-screen questionnaire and a nurse-led interview. We defined a healthy lifestyle as never smoking, moderate alcohol consumption, a healthy diet, regular physical activity, a normal BMI (18.5–24.9kg/m^2^), and healthy sleep duration. Specifically, we defined moderate alcohol consumption as 0–14 g/day for females and 0–28 g/day for males. We defined regular physical activity as at least 150 minutes of moderate activity per week or 75 minutes of vigorous activity per week (or an equivalent combination), or having moderate physical activity at least five days a week or vigorous activity once a week, as recommended by the American Heart Association [21]. We defined adequate sleep duration as 7.0–8.0 hours/day [22]. Furthermore, we defined diet pattern based on the following dietary components: fruits ≥3 servings/d; vegetables ≥3 servings/day; fish ≥2 servings/week; processed meats ≤1 serving/week; unprocessed red meats ≤1.5 servings/week; whole grains ≥3 servings/day; refined grains ≤1.5 servings/day [23]. We defined a healthy diet as a score ≥4. Participants were assigned 1 point for each healthy lifestyle behaviour, with a higher lifestyle score indicating adherence to healthy lifestyle (0–6 score) [16]. We categorised all participants into three lifestyle groups: unhealthy (0–2 score), intermediate (3–4 score), and healthy (5–6 score).

### Joint effect of SES and healthy lifestyle score on incident IBD risk

To further assess the joint effect of SES and healthy lifestyle score on risk of incident IBD, UC and CD, we categorised the participants into nine groups based on SES patterns (low, medium and high)/SES scores (0–2, 3–4 and 5–6) and healthy lifestyle scores (0–2, 3–4, and 5–6), with the low SES pattern/SES score of 0–2 and healthy lifestyle score of 0–2 as the reference group.

### Outcome ascertainment

The primary outcome was incident IBD, which was identified through linkage to primary care and hospital inpatient data using relevant ICD codes (K51 for UC and K50 for CD). Secondary outcomes included incident UC and CD. Participants who were diagnosed with both UC and CD were defined as IBD unclassified. Owing to the limited number of IBD unclassified cases, we combined IBD unclassified with UC or CD when separately evaluating the associated risk of UC or CD. Participants were followed from baseline until the date of IBD diagnosis, death, or the end of follow-up (30 September 2023), whichever came first.

### Covariates

Based on prior epidemiological evidence and data availability [16,24,25], potential confounders at baseline included age (continuous), sex (male, female), ethnicity (White, non-White), study centre (England, Scotland, Wales), depression (yes, no), anxiety (yes, no), and type 2 diabetes (T2DM) (yes, no). We used ICD-10 codes to determine depression (F32), anxiety (F40 and F41), and T2DM (E11). We also categorised the Townsend Deprivation Index (TDI), a measure of area-based socioeconomic deprivation, into quartiles and adjusted for it in sensitivity analyses. Missing data were limited to ethnicity (0.25%), with all other analytical variables complete; therefore, we created a missing-indicator category for ethnicity to retain the full analytical sample.

### Statistical analysis

We described baseline characteristics as mean and standard deviation for continuous variables (normal distribution) and as counts and proportions for categorical variables. We compared baseline characteristics among groups using the analysis of variance for continuous variables (normal distribution) and the χ^2^ test for categorical variables. We calculated the IBD incidence rate per 100,000 person-years with 95% confidence intervals (CI) using Poisson regression.

Cox proportional hazards models were used to estimate hazard ratios (HRs) and 95% CIs for the associations between SES pattern, SES scores, individual SES components, and the risk of incident IBD, UC, and CD. In addition to the crude model, we constructed two adjustment models: model 1 adjusted for age and sex; model 2 additionally adjusted for ethnicity, study centre, depression, anxiety, and T2DM. Given the very small percentage of missing values for most variables, we used missing indicators. We calculated the *P* for trend using values of 0 for low, 1 for medium, and 2 for high SES patterns, and the median value for each SES category.

We also performed mediation analysis using the SAS CAUSALMED procedure to estimate HRs for the natural direct effect and natural indirect effect of SES and its individual components on incident IBD, as well as the proportion of the association mediated by healthy lifestyle score (continuous). Potential confounders, including age, sex, ethnicity, study centre, depression, anxiety, and T2DM, were controlled for in the mediation analysis. Furthermore, for each SES component, we accounted for the other two components. This analysis was grounded in the hypothesis that SES shapes IBD risk in part through its upstream influence on lifestyle behaviours, whereby higher SES facilitates the adoption of healthier lifestyles, which in turn reduces IBD susceptibility, positioning healthy lifestyle score as a causally intermediate variable in the SES–IBD pathway.

We used a directed acyclic graph to illustrate the hypothesised causal relationships among SES, lifestyle factors, confounders, and incident IBD (Figure S3 in the **Online Supplementary Document**).

Furthermore, for SES patterns, we conducted subgroup analyses of SES scores and each SES component by age (<60, ≥60 years) and sex (male, female). Potential effect modification was tested by adding interaction terms of each stratified variable and each SES exposure *via* adjusted model 2. We performed several sensitivity analyses to test the robustness of our findings, including: excluding IBD/UC/CD cases diagnosed within 2, 3 or 5 years after baseline to minimise reverse causation; additionally adjusting for TDI quartiles; using competing risk models with death as a competing event; excluding participants who died during follow-up; excluding individuals with prevalent diabetes, depression, and anxiety at baseline; and using age-scaled Cox regression stratified by five-year birth cohort. To assess robustness to unmeasured confounding, we calculated an E-value for each non-reference category in model 2.

We considered a two-tailed *P*-value <0.05 statistically significant. We used SAS, version 9.4 (SAS Institute Inc., Cary, North Carolina, USA) and *R*, version 4.3.1 (R Core Team, Vienna, Austria) for all analyses.

## RESULTS

### Baseline characteristics

We included 374,507 participants with a mean age of 55.84 years (standard deviation = 8.09), of whom 51.5% were female (**Table 1**). Of these, 76,292 (20.4%) were classified as high SES, 197,130 (52.6%) as medium, and 101,085 (27.0%) as low SES. Compared to participants with a low SES pattern, those with a high SES pattern were more likely to be younger, male, never smoking, maintaining a healthy diet, engaging in regular physical activity, having a normal BMI, achieving healthy sleep duration, and having a lower prevalence of depression, anxiety, and T2DM.

**Table 1:** Baseline characteristics of all participants by SES

	Total (n = 374,507)	High SES (n = 76292)	Medium SES (n = 197130)	Low SES (n = 101085)	*P*-value
**Age in years, x̄ (SD)**	55.84 (8.09)	52.29 (7.15)	55.48 (8.00)	59.20 (7.62)	<0.001
**Sex**					<0.001
Male	181,795(48.5)	39,542(51.8)	96,966(49.2)	45,287(44.8)	
Female	192,712 (51.5)	36,750 (48.2)	100,164 (50.8)	55,798 (55.2)	
**Study centre**					<0.001
England	331,037 (88.4)	66,954 (87.8)	174,442 (88.5)	89,641 (88.7)	
Scotland	27,589 (7.4)	6,507 (8.5)	13,953 (7.1)	7,129 (7.1)	
Wales	15,881 (4.2)	2,831 (3.7)	8,735 (4.4)	4,315 (4.3)	
**Ethnicity**					<0.001
Non-White	18,193 (4.9)	3,111 (4.1)	8,729 (4.4)	6,353 (6.3)	
White	355,390 (94.9)	73,033 (95.7)	187,932 (95.3)	94,425 (93.4)	
Unknown	924 (0.2)	148 (0.2)	469 (0.2)	307 (0.3)	
**Household income (GBP)**					<0.001
<18,000	81,310 (21.7)	0 (0.0)	0 (0.0)	81,310 (80.4)	
18,000–30,999	94,079 (25.1)	0 (0.0)	75,292 (38.2)	18,787 (18.6)	
31,000–51,999	99,272 (26.5)	1,076 (1.4)	97,799 (49.6)	397 (0.4)	
52,000–100,000	78,784 (21.0)	54,558 (71.5)	24,039 (12.2)	187 (0.2)	
>100,000	21,062 (5.6)	20,658 (27.1)	0 (0.0)	404 (0.4)	
**Education level**					<0.001
College or above	133,115 (35.5)	59,441 (77.9)	60,125 (30.5)	13,549 (13.4)	
High school or equivalent	187,693 (50.1)	16,851 (22.1)	128,015 (64.9)	42,827 (42.4)	
Less than high school	53,699 (14.3)	0 (0.0)	8,990 (4.6)	44,709 (44.2)	
**Employment status**					<0.001
Employed, student, or retired	348,854 (93.2)	71,541 (93.8)	194,926 (98.9)	82,387 (81.5)	
Unemployed	25,653 (6.8)	4,751 (6.2)	2,204 (1.1)	18,698 (18.5)	
**TDI, x̄ (SD)**	−1.35 (3.05)	−2.02 (2.69)	−1.70 (2.80)	−0.16 (3.41)	<0.001
**TDI**					<0.001
Q1 (≤−3.63)	93,517 (25.0)	24,462 (32.1)	53,551 (27.2)	15,504 (15.4)	
Q2 (−3.63, −2.11)	93,512 (25.0)	20,066 (26.3)	52,852 (26.8)	20,594 (20.4)	
Q3 (−2.11, 0.58)	93,488 (25.0)	18,054 (23.7)	49,926 (25.4)	25,508 (25.3)	
Q4 (>0.58)	93,500 (25.0)	13,619 (17.9)	40,529 (20.6)	39,352 (39.0)	
**Healthy lifestyle scores**					<0.001
0–2	111,831 (29.9)	28,100 (36.8)	59,846 (30.4)	23,885 (23.6)	
3–4	203,327 (54.3)	39,361 (51.6)	107,504 (54.5)	56,462 (55.9)	
5–6	59,349 (15.8)	8,831 (11.6)	29,780 (15.1)	20,738 (20.5)	
**Healthy lifestyle factors**					
Never smoking	205,840 (55.0)	47,694 (62.5)	110,186 (55.9)	47,960 (47.4)	<0.001
Moderate alcohol drinking	236,448 (63.1)	45,989 (60.3)	125,910 (63.9)	64,549 (63.9)	<0.001
Healthy diet	272,445 (72.7)	59,519 (78.0)	143,719 (72.9)	69,207 (68.5)	<0.001
Regular physical activity	292,070 (78.0)	61,602 (80.7)	155,516 (78.9)	74,952 (74.1)	<0.001
Normal BMI	121,999 (32.6)	30,454 (39.9)	63,654 (32.3)	27,891 (27.6)	<0.001
Healthy sleep duration	256,828 (68.6)	57,224 (75.0)	138,285 (70.1)	61,319 (60.7)	<0.001
Depression	30,579 (8.2)	4,239 (5.6)	14,439 (7.3)	11,901 (11.8)	<0.001
Anxiety	14,152 (3.8)	1,985 (2.6)	6,878 (3.5)	5,289 (5.2)	<0.001
T2DM	9,066 (2.4)	847 (1.1)	3,879 (2.0)	4,340 (4.3)	<0.001

SES – socioeconomic status, SD – standard deviation, TDI – Townsend Deprivation Index, T2DM – type 2 diabetes, x̄ – mean

*Values are presented as n (%) unless specified otherwise.

### SES pattern, SES scores and individual SES components with risk of incident IBD

Over a median of 14.57 years' (5,306,265 person-years) follow-up, 2381 incident cases of IBD were identified, including 1703 cases of UC and 843 cases of CD (**Table 2**). After adjusting for multiple covariates, individuals with high SES (HR = 0.55; 95% CI = 0.48–0.63; absolute risk reduction = 27.3 per 100,000 person-years) and medium SES (HR = 0.80; 95% CI = 0.73–0.88; absolute risk reduction = 12.8 per 100,000 person-years) demonstrated a significantly reduced risk of developing IBD compared to those with low SES. Compared to individuals with SES scores of 0–2, those with scores of 3–4 (HR = 0.75; 95% CI = 0.67–0.85) and 5–6 (HR = 0.58; 95% CI = 0.52–0.66) had a reduced risk of IBD. The trend across categories of both SES patterns and scores was statistically significant (*P* for trend <0.001). Moreover, per 1 SES score increment was associated with a 13% reduction in IBD risk (HR = 0.86; 95% CI = 0.83–0.88). The protective association of high SES pattern was consistent for UC (HR = 0.54; 95% CI = 0.46–0.64) and CD (HR = 0.52; 95% CI = 0.41-0.65), similar to higher SES score (5–6) for both UC (HR = 0.58; 95% CI = 0.50–0.67) and CD (HR = 0.53; 95% CI = 0.43–0.66).

**Table 2:** Risk of incident IBD, UC, and CD associated with SES*

				HR (95%CI)			
	**Events, n**	**Participants, n**	**Person-years**	**Crude**	**Model 1**	**Model 2**	**E-value point (CI bound)**
**Incident IBD**							
SES pattern							
*Low SES*	802	101,085	1,400,468	ref	ref	ref	
*Medium SES*	1,250	197,130	2,808,779	0.78 (0.71–0.85)	0.79 (0.72–0.86)	0.80 (0.73–0.88)	1.81 (1.53)
*High SES*	329	76,292	1,097,017	0.52 (0.46–0.60)	0.54 (0.47–0.61)	0.55 (0.48–0.63)	3.04 (2.55)
*P for trend*				<0.001	<0.001	<0.001	
SES score, categorical							
*0-2*	392	43,529	595,926	ref	ref	ref	
*3–4*	1,196	177,636	2,513,610	0.72 (0.65–0.81)	0.74 (0.66–0.83)	0.75 (0.67–0.85)	2.00 (1.63)
*5–6*	793	153,342	2,196,729	0.55 (0.49–0.62)	0.56 (0.50–0.64)	0.58 (0.52–0.66)	2.84 (2.40)
*P for trend*				<0.001	<0.001	<0.001	
SES score, continuous	2,381	374,507	5,306,265	0.84 (0.82–0.87)	0.85 (0.82–0.87)	0.86 (0.83–0.88)	1.60 (1.53)
**Incident UC**							
SES pattern							
*Low SES*	564	101,085	1,400,468	ref	ref	ref	
*Medium SES*	914	197,130	2,808,779	0.81 (0.73–0.90)	0.82 (0.74–0.91)	0.84 (0.75–0.93)	1.67 (1.36)
*High SES*	225	76,292	1,097,017	0.51 (0.44–0.60)	0.53 (0.45–0.62)	0.54 (0.46–0.64)	3.11 (2.50)
*P for trend*				<0.001	<0.001	<0.001	
SES score, categorical							
0–2	278	43,529	595,926	ref	ref	ref	
3–4	866	177,636	2,513,610	0.74 (0.65–0.85)	0.75 (0.66–0.86)	0.77 (0.67–0.88)	1.92 (1.53)
5–6	559	153,342	2,196,729	0.55 (0.47–0.63)	0.56 (0.49–0.65)	0.58 (0.50–0.67)	2.84 (2.35)
*P for trend*				<0.001	<0.001	<0.001	
SES score, continuous	1,703	374,507	5,306,265	0.84 (0.82–0.87)	0.85 (0.82–0.88)	0.86 (0.83–0.89)	1.60 (1.50)
**Incident CD**							
SES pattern							
*Low SES*	302	101,085	1,400,468	ref	ref	ref	
*Medium SES*	421	197,130	2,808,779	0.70 (0.60–0.81)	0.69 (0.59–0.80)	0.71 (0.61–0.82)	2.17 (1.74)
*High SES*	120	76,292	1,097,017	0.51 (0.41–0.63)	0.49 (0.40–0.62)	0.52 (0.41–0.65)	3.26 (2.45)
*P for trend*				<0.001	<0.001	<0.001	
SES score, categorical							
*0–2*	148	43,529	595,926	ref	ref	ref	
*3–4*	419	177,636	2,513,610	0.67 (0.56–0.81)	0.67 (0.56–0.81)	0.70 (0.58–0.84)	2.21 (1.67)
*5–6*	276	153,342	2,196,729	0.51 (0.42–0.62)	0.51 (0.41–0.62)	0.53 (0.43–0.66)	3.18 (2.40)
*P for trend*				<0.001	<0.001	<0.001	
SES score, continuous	843	374,507	5,306,265	0.83 (0.79–0.87)	0.83 (0.79–0.87)	0.84 (0.80–0.88)	1.67 (1.53)

CD – Crohn’s disease, CI – confidence interval, HR – hazard ratio, IBD – inflammatory bowel disease, SES – socioeconomic status, UC – ulcerative colitis, ref – reference

*Model 1 was adjusted for age and sex; model 2 was adjusted for age, sex, ethnicity, study centre, depression, anxiety and type 2 diabetes. *P* for trend was calculated using 0 for low, 1 for medium, and 2 for high SES, and the median value for each SES category.

Individuals with high (HR = 0.63; 95% CI = 0.56–0.72) and medium (HR = 0.82; 95% CI = 0.75–0.91) income had a lower IBD risk than those with low income (**Table 3**). Education level showed the strongest beneficial effect, with college or above (HR = 0.58; 95% CI = 0.51–0.65) and high school education (HR = 0.77; 95% CI = 0.69–0.85) associated with a lower IBD risk than those with less than high school education. Employed, student, or retired individuals had a 29% (HR = 0.71; 95% CI = 0.61–0.82) lower IBD risk than unemployed participants. Similar findings were observed for UC and CD across all socioeconomic factors, with a slightly stronger effect for CD than UC. Notably, higher education showed the strongest protective effect against both UC (HR = 0.56; 95% CI = 0.49–0.65) and CD (HR = 0.56; 95% CI = 0.45–0.68). All trends across categories of income and education were statistically significant (*P* for trend <0.001).

**Table 3:** Risk of incident IBD, UC, and CD associated with each item of SES*

				HR (95%CI)		
	**Events, n**	**Participants, n**	**Person-years**	**Crude**	**Model 1**	**Model 2**
**Incident IBD**						
Household income						
*Low*	642	81,310	1,122,895	ref	ref	ref
*Medium*	1,249	193,351	2,748,575	0.80 (0.72–0.88)	0.80 (0.73–0.89)	0.82 (0.75–0.91)
*High*	490	99,846	1,434,794	0.60 (0.53–0.67)	0.61 (0.54–0.69)	0.63 (0.56–0.72)
*P for trend*				<0.001	<0.001	<0.001
Education level						
*Less than high school*	476	53,699	743,229	ref	ref	ref
*High school or equivalent*	1,240	187,693	2,662,202	0.73 (0.65–0.81)	0.76 (0.68–0.85)	0.77 (0.69–0.85)
*College or above*	665	133,115	1,900,834	0.55 (0.49–0.61)	0.57 (0.51–0.65)	0.58 (0.51–0.65)
*P for trend*				<0.001	<0.001	<0.001
Employment status						
*Unemployed*	216	25,653	355,907	ref	ref	ref
*Employed, student or retired*	2,165	348,854	4,950,358	0.72 (0.63–0.83)	0.68 (0.59–0.78)	0.71 (0.61–0.82)
**Incident UC**						
Household income						
*Low*	449	81,310	1,122,895	ref	ref	ref
*Medium*	911	193,351	2,748,575	0.83 (0.74–0.93)	0.84 (0.75–0.95)	0.86 (0.77–0.97)
*High*	343	99,846	1,434,794	0.60 (0.52–0.69)	0.62 (0.53–0.72)	0.64 (0.55–0.74)
*P for trend*				<0.001	<0.001	<0.001
Education level						
*Less than high school*	344	53,699	743,229	ref	ref	ref
*High school or equivalent*	895	187,693	2,662,202	0.73 (0.64–0.82)	0.77 (0.68–0.87)	0.77 (0.68–0.88)
*College or above*	464	133,115	1,900,834	0.53 (0.46–0.61)	0.56 (0.48–0.65)	0.56 (0.49–0.65)
*P for trend*				<0.001	<0.001	<0.001
Employment status						
*Unemployed*	149	25,653	355,907	ref	ref	ref
*Employed, student or retired*	1,554	348,854	4,950,358	0.75 (0.64–0.89)	0.70(0.59–0.83)	0.72(0.61–0.86)
**Incident CD**						
Household income						
*Low*	243	81,310	1,122,895	ref	ref	ref
*Medium*	429	193,351	2,748,575	0.72 (0.62–0.85)	0.72 (0.61–0.84)	0.74 (0.63–0.87)
*High*	171	99,846	1,434,794	0.55 (0.45–0.67)	0.54 (0.44–0.66)	0.57 (0.46–0.70)
*P for trend*				<0.001	<0.001	<0.001
Education level						
*Less than high school*	173	53,699	743,229	ref	ref	ref
*High school or equivalent*	429	187,693	2,662,202	0.69 (0.58–0.82)	0.69 (0.57–0.83)	0.70 (0.58–0.84)
*College or above*	241	133,115	1,900,834	0.54 (0.45–0.66)	0.54 (0.44–0.66)	0.56 (0.45–0.68)
*P for trend*				<0.001	<0.001	<0.001
Employment status						
Unemployed	84	25,653	355,907	ref	ref	ref
Employed, student or retired	759	348,854	4,950,358	0.65 (0.52–0.82)	0.63 (0.50–0.79)	0.68 (0.54–0.85)

CD – Crohn’s disease, CI – confidence interval, HR – hazard ratio, IBD – inflammatory bowel disease, SES – socioeconomic status, UC – ulcerative colitis, ref – reference

*Model 1 was adjusted for age, sex and the other two items of socioeconomic factors; Model 2 was adjusted for age, sex, ethnicity, study centre, depression, anxiety, type 2 diabetes and the other two items of socioeconomic factors. *P* for trend was calculated using 0 for low, 1 for medium, and 2 for high SES.

Subgroup analyses showed that the lower IBD risk associated with high SES was consistently observed across age and sex strata, with stronger effects among younger individuals (*P*-interaction <0.05). Similar patterns were noted for both UC and CD, with generally stronger associations for CD than UC (**Figure 1**). Similarly, the reduced risk of IBD, UC and CD associated with high household income, college or above, and employed status was generally observed across age and sex subgroups, particularly in those younger than 60 years (Tables S4 and S5 in the **Online Supplementary Document**).

**Figure 1:**
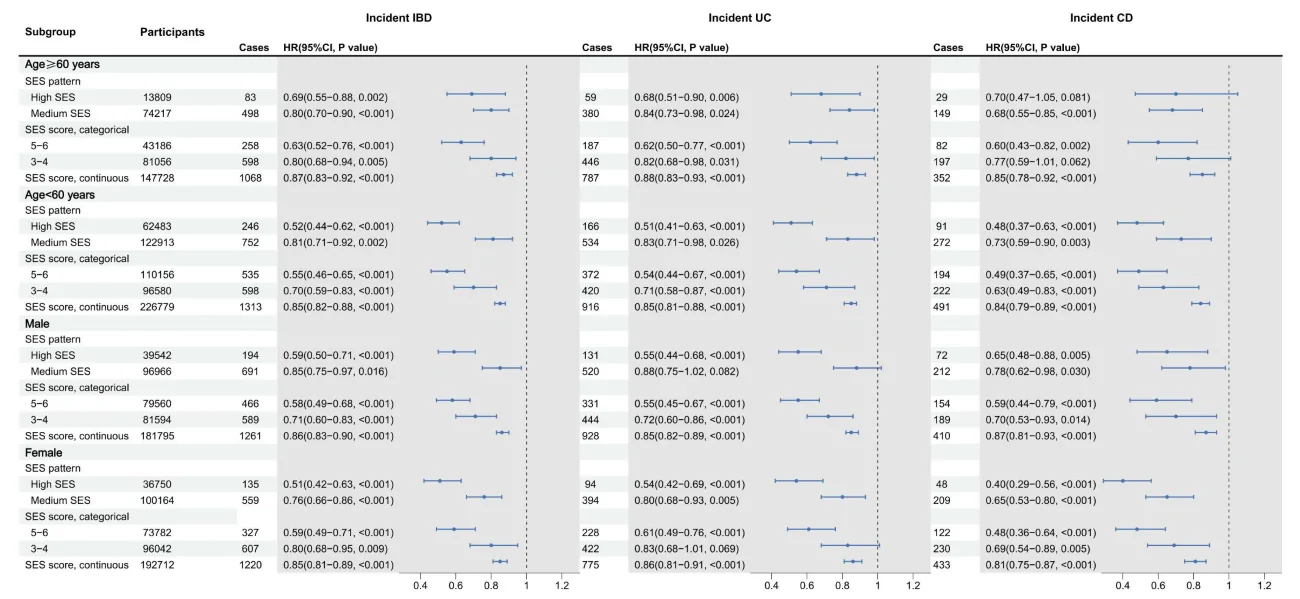
Subgroup analysis of SES status with risk of incident IBD, UC, and CD by age and sex. CD – Crohn’s disease, CI – confidence interval, HR – hazard ratio, IBD – inflammatory bowel disease, SES – socioeconomic status, UC – ulcerative colitis. All HRs were adjusted for age, sex, ethnicity, study centre, depression, anxiety and type 2 diabetes.

### Joint associations of SES and healthy lifestyle with incident IBD

Joint analysis demonstrated a lower IBD risk associated with higher SES pattern or score across all lifestyle categories (**Figure 2**, Panels A–F; Tables S6 and S7 in the **Online Supplementary Document**). Among participants with the healthiest lifestyle score (5–6), those with a high SES pattern (HR = 0.37; 95% CI = 0.29–0.46) had a lower IBD risk compared to those with low SES. When analysed by SES scores, individuals with both a high SES score (5–6) and a healthy lifestyle score (5–6) (HR = 0.32; 95% CI = 0.26–0.39) had lower IBD risk compared with those with low SES scores and a low lifestyle score. Similar findings were observed for both UC and CD risk, with generally stronger associations for CD.

**Figure 2:**
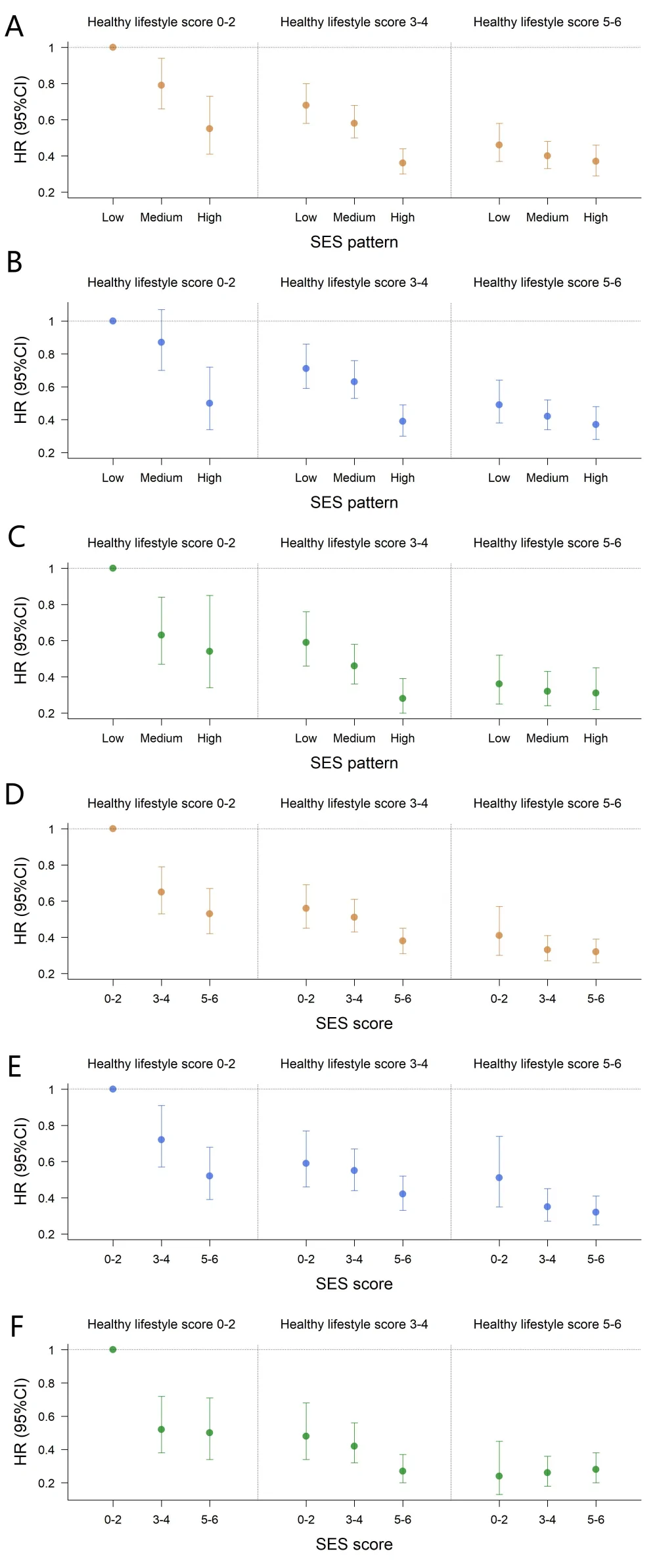
Joint associations of SES pattern/score and healthy lifestyle score with incident IBD, UC and CD. **Panel A.** SES pattern, healthy lifestyle, and incident IBD. **Panel B.** SES pattern, healthy lifestyle score, and incident UC. **Panel C.** SES pattern, healthy lifestyle, and incident CD. **Panel D.** SES score, healthy lifestyle, and incident IBD. **Panel E.** SES score, healthy lifestyle, and incident UC. **Panel F.** SES score, healthy lifestyle, and incident CD. All HRs were adjusted for age, sex, ethnicity, study centre, depression, anxiety and type 2 diabetes. CD – Crohn’s disease, CI – confidence interval, HR – hazard ratio, IBD – inflammatory bowel disease, SES – socioeconomic status, UC – ulcerative colitis.

Subgroup analyses showed that the joint protective association of high SES and a healthy lifestyle against IBD risk was consistent across age and sex subgroups, with stronger effects among participants younger than 60 years (*P*-interaction <0.05) and females (*P*-interaction <0.05) (Tables S8–11 in the **Online Supplementary Document**).

### Mediation analyses of healthy lifestyle factors on SES-IBD association

Mediation analyses indicated that lifestyle factors partially mediated the association between SES and IBD risk (**Figure 3**, Panels A–C). For the comparison between high *vs.* low SES pattern, the natural direct effect (HR = 0.62; 95% CI = 0.53–0.71) and natural indirect effect through lifestyle factors (HR = 0.92; 95% CI = 0.90–0.94) together contributed to the total protective effect, with lifestyle factors mediating 11.34% (6.47–16.22%) of the association. For the comparison between the highest SES score (5–6) and the lowest (0–2), lifestyle factors mediated 14.91% (8.68–21.14%) of the total protective association. Furthermore, lifestyle factors accounted for 16.50% (11.81–21.20%) of the association mediated per 1 SES score.

**Figure 3:**
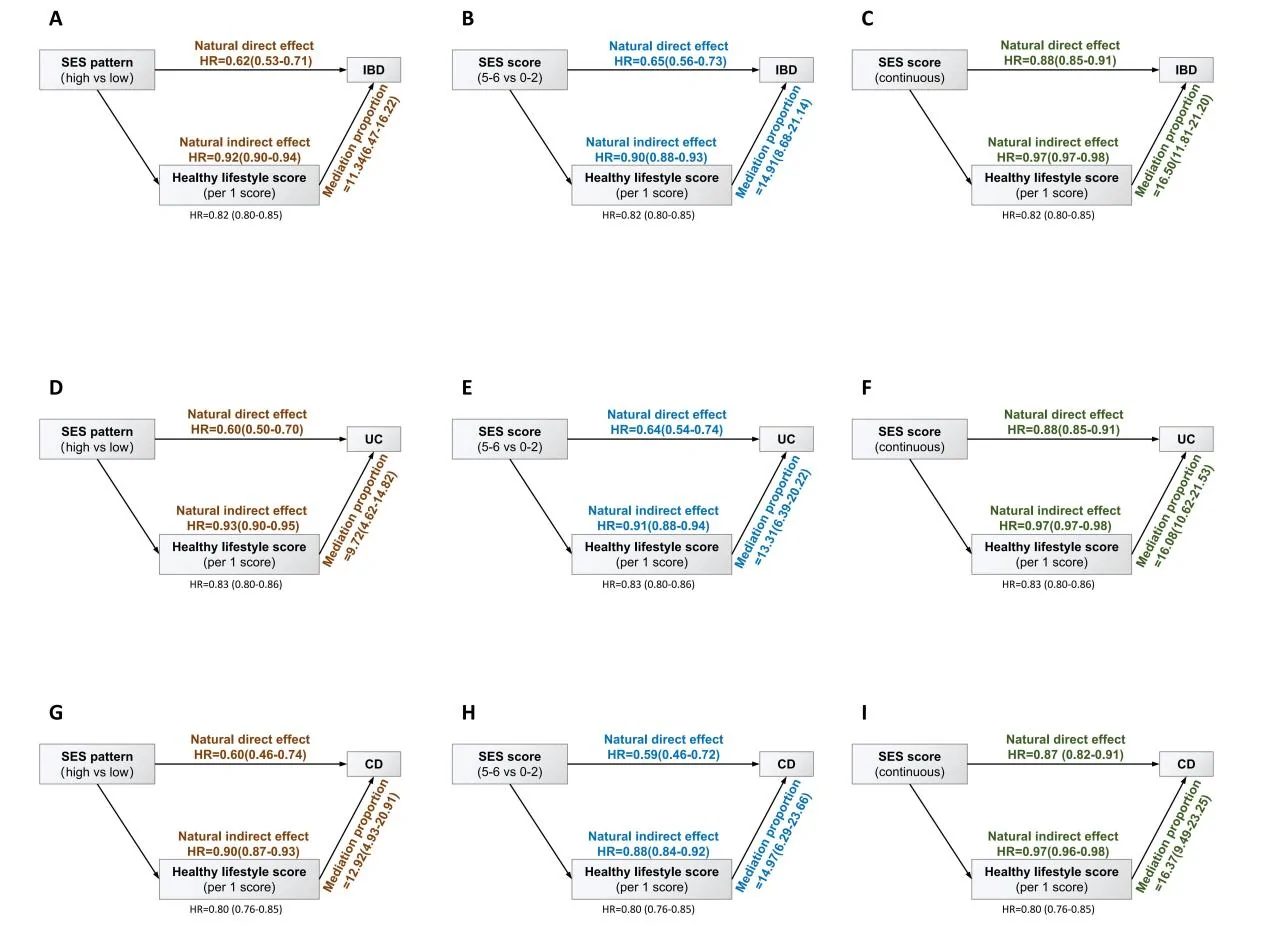
Natural direct effect of incident IBD, UC, and CD associated with SES and the proportion mediated by healthy lifestyle score. **Panel A.** SES pattern (high *vs.* low), healthy lifestyle score, and IBD. **Panel B.** SES score (5–6 *vs.* 0–2), healthy lifestyle score, and IBD. **Panel C.** SES score (continuous), healthy lifestyle score, and IBD. **Panel D.** SES pattern (high *vs.* low), healthy lifestyle score, and UC. **Panel E.** SES score (5–6 *vs.* 0–2), healthy lifestyle score, and UC. **Panel F.** SES score (continuous), healthy lifestyle score, and UC. **Panel G.** SES pattern (high *vs.* low), healthy lifestyle score, and CD. **Panel H.** SES score (5–6 *vs.* 0–2), healthy lifestyle score, and CD. **Panel I.** SES score (continuous), healthy lifestyle score, and CD. All HRs were adjusted for age, sex, ethnicity, study centre, depression, anxiety and type 2 diabetes. CD – Crohn’s disease, CI – confidence interval, HR – hazard ratio, IBD – inflammatory bowel disease, SES – socioeconomic status, UC – ulcerative colitis.

The mediation effect was consistent across IBD subtypes. For UC, lifestyle factors mediated 9.72% (4.62–14.82%) of the association between high *vs.* low SES pattern, 13.31% (6.39–20.22%) for highest *vs.* lowest SES score, and 16.08% (10.62–21.53%) per 1 SES score increment (**Figure 3**, Panels D–F). For CD, lifestyle factors mediated 12.92% (4.93–20.91%) of the association between high *vs* low SES pattern, 14.97% (6.29–23.66%) for highest *vs.* lowest SES score, and 16.37% (9.49–23.25%) per 1 SES score increment (**Figure 3**, Panels G–I).

### Sensitivity analysis

The sensitivity analyses showed consistently lower risks of IBD, UC and CD associated with higher SES when excluding IBD cases diagnosed within two or three years after baseline, using competing risk models, excluding participants who died during follow-up, excluding individuals with prevalent diabetes, depression and anxiety at baseline, additionally adjusting for TDI quartiles, or using age-scaled Cox regression stratified by five-year birth cohort (Tables S12–19 in the **Online Supplementary Document**).

## DISCUSSION

In this large prospective cohort study of 374,507 participants from the UKB, we found a significant association between SES and IBD risk. Specifically, individuals with a high SES pattern had a 45% lower risk of developing IBD than those with low SES, with consistent protective effects for both UC and CD. All components of SES, including high household income, high education level, and employed status, were also associated with reduced IBD risk, with high education demonstrating the strongest protective effect. Furthermore, healthy lifestyle factors partially mediated the favourable effect of high SES pattern and score, accounting for 11.34–16.50% of the total effect. Individuals with both high SES and a healthy lifestyle score had an additional reduced risk by 63% for IBD, 63% for UC, and 69% for CD. These findings suggest the pivotal role of socioeconomic inequity in reducing the disease burden of IBD. However, considering the observational nature of our study, these associations should not be interpreted as causal without further experimental or quasi-experimental evidence.

Interestingly, previously published studies have shown conflicting results on the association between SES and IBD risk due to heterogeneity in study design and population. In recently reported cross-sectional studies, a higher prevalence of IBD was observed in high-income, urban areas or newly developed countries [1]. The previous ‘hygiene hypothesis’ proposed that individuals with high SES, living in more hygienic environments, might experience reduced childhood exposure to common pathogens, potentially leading to abnormal immune responses in genetically susceptible individuals and thereby increasing IBD risk [5,6]. However, several population-based studies reported the beneficial effect of higher income and higher education level on IBD risk, which was consistent with our study [9]. Another case-control study found that infants from low-SES families had a greater risk of developing IBD compared to those from high-SES families (odds ratio = 3.0; 95% CI = 1.5–6.1) [26]. Additionally, a French cohort study using national health insurance databases also reported higher CD incidence correlated with higher unemployment and poverty rates [10].

Multiple potential mechanisms may explain the relationship between higher SES and lower IBD risk. First, individuals with higher SES typically have better access to healthcare, including preventive screenings and early medical interventions, which may help reduce IBD development [27]. Notably, although the UK National Health Service provides universal healthcare coverage, SES-related disparities in healthcare utilisation persist, as low-SES individuals may face longer waiting times, lower health literacy, and reduced capacity to navigate complex healthcare pathways, potentially delaying IBD diagnosis and early intervention. Second, low-SES populations are often exposed to chronic psychosocial stress, which may dysregulate the hypothalamic-pituitary-adrenal axis, promote pro-inflammatory cytokine release, and alter gut microbiota composition, collectively increasing intestinal permeability and mucosal immune activation in genetically susceptible individuals [2,8,28]; greater exposure to environmental pollutants common in low-SES communities may further amplify these inflammatory pathways [11]. Beyond lifestyle behaviours, chronic psychosocial stress associated with socioeconomic adversity may dysregulate the hypothalamic-pituitary-adrenal axis, alter gut microbiota composition, and increase intestinal permeability, collectively facilitating mucosal immune dysregulation in genetically susceptible individuals. Furthermore, emerging evidence suggests that early-life socioeconomic adversity may programme innate immune responses through epigenetic modifications, potentially elevating IBD risk independently of adult lifestyle choices [29]. Moreover, higher educational attainment is often linked to improved health awareness and decision-making, which may enable individuals to manage their health more effectively and reduce exposure to IBD risk factors. Nevertheless, given the observational nature of our study, the underlying mechanisms remain unclear, and further investigation is warranted to understand the complex interplay between SES and IBD risk.

Furthermore, we also found that healthy lifestyle factors accounted for approximately 11–16% of the association between high SES and reduced IBD risk. This finding aligns with previous research on lifestyle factors as mediators between SES and other health outcomes [12,13,30]. A systematic review of 31 studies [14] reported that lifestyle factors could explain approximately 20–30% of SES inequities in health outcomes. Although the mediation proportion in our study is relatively modest, it still highlights the importance of healthy lifestyle factors in reducing the effect of SES disparities on adverse outcomes, with a 59% reduced IBD risk even among individuals with low SES by maintaining a healthy lifestyle. Meanwhile, the protective effect of healthy lifestyle was more pronounced in low-SES populations, emphasising the need for targeted lifestyle interventions, particularly among those vulnerable to adverse health behaviours [14]. Nevertheless, the substantial proportion of the SES-IBD association unexplained by lifestyle factors suggests that other pathways are likely operative, including chronic psychosocial stress, greater exposure to environmental pollutants, and differential access to preventive healthcare, all of which disproportionately burden low-SES individuals and may collectively promote intestinal immune dysregulation. Future studies should systematically investigate these complementary mechanisms to develop more comprehensive prevention strategies.

Moreover, UC and CD differ in underlying pathophysiology and environmental risk factor profiles, with CD more consistently associated with smoking and dietary composition, whereas UC shows a distinct, and at times opposing, relationship with smoking. Nonetheless, consistent with Sun *et al.* [16] who applied the same composite healthy lifestyle construct across IBD subtypes and reported broadly comparable protective associations for both UC and CD, we considered the overall healthy lifestyle pattern to exert a similar directional effect across subtypes within our mediation framework, while recognising that subtype-specific mechanisms may still differ.

Our findings have profound implications for clinical practice and policy. The observed associations were statistically robust and clinically meaningful. The absolute risk reduction of 27.3 per 100,000 person-years for high *vs.* low SES indicates that, at the population level, a considerable number of IBD cases could potentially be averted; in a population of one million individuals, this corresponds to approximately 273 fewer cases annually – representing substantial relief for patients, clinicians, and healthcare systems bearing the long-term burden of this chronic disease. The strong link between healthy lifestyle choices and a lower incidence of IBD highlights the potential of targeted public health interventions to promote healthier behaviours, particularly among individuals with low SES. The finding that lifestyle factors partially mediate the association between SES and IBD risk provides actionable targets for interventions to reduce socioeconomic health disparities. Therefore, addressing socioeconomic barriers by improving access to healthcare and education may enhance early detection and treatment adherence and improve overall prognosis, ultimately alleviating the disease burden in vulnerable populations and carrying profound implications for global health equity and IBD prevention policy, particularly in low- and middle-income countries experiencing a growing IBD burden.

To the best of our knowledge, this is the first large-scale prospective cohort study to comprehensively investigate the effect of SES on long-term incident IBD risk, using multiple indicators (income, education, and employment) as a composite SES measure, enabling a more holistic examination of SES influences on IBD risk. Furthermore, we conducted extensive mediation analyses to quantify the contribution of lifestyle factors to the SES-IBD relationship, offering insights into potential intervention targets. We also performed multiple sensitivity and subgroup analyses to test the robustness of our findings.

Several potential limitations should also be considered. First, the UKB cohort has a relatively high proportion of high-SES individuals and white participants of European ancestry, which may limit the generalisability of our findings to more diverse populations. Caution is warranted when extrapolating these findings to Africa, Latin America, South Asia, and other regions experiencing emerging IBD epidemics, where distinct genetic backgrounds, infectious disease burdens, dietary patterns, and healthcare infrastructures may substantially modify the SES-IBD relationship. Meanwhile, the UKB is subject to healthy volunteer bias, as participants are generally healthier, more educated, and of higher SES than the general population, which may have attenuated the observed SES-IBD gradient and limited generalisability. Selection bias cannot be ruled out, as lower-SES individuals are less likely to participate in long-term cohort studies. Furthermore, IBD diagnoses were ascertained through linked health records, raising the possibility of differential diagnostic ascertainment across SES groups, since higher-SES individuals may access specialist care and receive formal diagnoses more readily than their lower-SES counterparts. Second, lifestyle factors were assessed only at baseline, precluding examination of dynamic changes in lifestyle behaviours over time and their impact on the SES-IBD relationship. Further longitudinal assessments of lifestyle factors may provide more comprehensive insights into the mechanisms linking SES to IBD risk. Third, SES components and status were collected only at baseline. However, they may vary over the long-term follow-up period, potentially biasing the association. Additionally, IBD cases were identified through primary care and hospital inpatient data, which might have missed milder cases, potentially leading to underestimation of IBD incidence. Also, employment status, as one component of the SES construct, may be susceptible to reverse causation, as undiagnosed or early symptomatic IBD could itself influence an individual's capacity to remain employed; however, sensitivity analyses excluding IBD cases diagnosed within two, three, and five years of baseline yielded results consistent with the primary findings, mitigating this concern. Finally, given the observational nature of our study, we could not entirely eliminate residual confounding, although our prospective design, temporal sequence, and consistent findings across multiple analyses strengthen the likelihood of a causal relationship. However, even in the presence of potential unmeasured confounders, we calculated E-values to quantitatively assess robustness; the point estimate E-values ranged from 1.60 to 3.26, and the corresponding CI-bound E-values ranged from 1.36 to 2.55, indicating that a fairly substantial unmeasured confounder would be required to explain away these associations and supporting the robustness of our findings. Furthermore, we combined lifestyle factors using an unweighted scoring approach, which may not fully reflect the differential contribution of individual behaviours to IBD risk; future studies using weighted or data-driven scoring methods may offer more nuanced insights. Meanwhile, the mediation analyses should be interpreted with caution, as they rely on assumptions of no unmeasured confounding between the mediators and outcome that are unlikely to be fully satisfied in an observational setting; the estimated mediated proportions therefore reflect statistical decomposition rather than confirmed causal mechanisms.

## CONCLUSIONS

We found that high SES is significantly associated with reduced IBD risk, with high SES associated with a 45% reduction in IBD risk and medium SES with a 20% reduction compared to low SES, and healthy lifestyle factors statistically explained part of the observed association, accounting for approximately 11–16% of the total SES-IBD relationship. These findings emphasise the important roles of socioeconomic determinants and lifestyle behaviours in IBD pathogenesis. Public health interventions that promote healthy lifestyle behaviours, particularly among socioeconomically disadvantaged individuals, may offer a practical, actionable strategy to reduce IBD risk at the population level. Future research should explore the underlying mechanisms linking SES to IBD risk and evaluate the effectiveness of lifestyle interventions targeting socioeconomically disadvantaged populations in reducing IBD risk.

## Additional material


Online Supplementary Document

## Data Availability

**Data availability:** We obtained all study-related data from the UKB Resource under application number 74444. No additional data are available.
