# Supplementary material for: Socioeconomic status and long-term risk of inflammatory bowel disease: a prospective cohort study with mediation analysis
Source: J Glob Health. 2026 Nov 6;16:04391. doi: 10.7189/jogh.16.04391 (PMC13741165; doi:10.7189/jogh.16.04391)
Supplement: Supplementary file 1 [file jogh-16-04391-s001.pdf]

Supplement to: Zhang Q, Wang Y, Yu S, Zhou Y, Liu Si, Zhang S, Li P, Wu S. Socioeconomic status and long-term risk of inflammatory bowel disease: a prospective cohort study with mediation analysis. J Glob Health. 2026;16:04391.

Figure S1. Flowchart of the study population

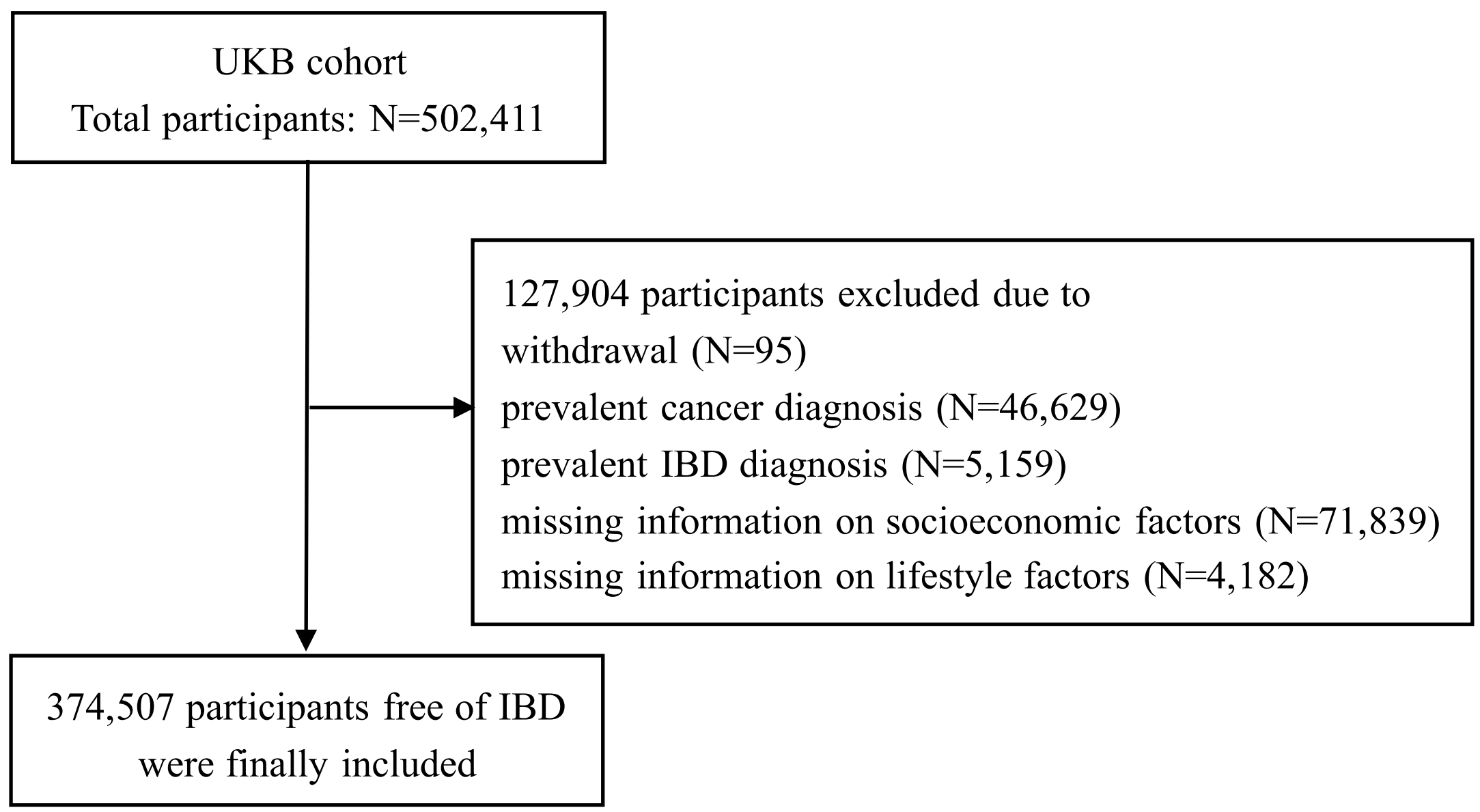

Note: IBD: inflammatory bowel disease; UKB: UK Biobank.

Supplementary Methods

Assessment of socioeconomic status using latent class analysis

Socioeconomic status (SES) was derived from average total household income before tax, education level, and employment status in the UK Biobank (UKB). Since the National Health Service in the UK provides comprehensive, universal, and free healthcare services, health insurance was not included as a component of SES. Participants were categorized into five groups based on their average total household income before tax: "less than £18,000", "£18,000 to £30,999", "£31,000 to £51,999", "£52,000 to £100,000", and "greater than £100,000". Education level were divided into seven categories: "College or University degree", "A levels/AS levels or equivalent", "O levels/GCSEs or equivalent", "CSEs or equivalent", "NVQ or HND or HNC or equivalent", "Other professional qualifications", and "None of the above" (equivalent to less than high school diploma). We did not re-classified household income and education level because of the sufficient sample size within each subgroup. Employment status, collected at baseline without specific occupational details, was categorized into two groups: employed (including "in paid employment or self-employed", "retired", "engaged in unpaid or voluntary work", and "studying full or part-time") and unemployed (including "looking after home and/or family", "unable to work due to sickness or disability", and "unemployed").

We utilized Latent Class Analysis (LCA) via the R package *poLCA* (v1.6.0) to estimate SES based on the aforementioned three variables. The maximum number of iterations and the tolerance value for determining convergence were set to 10,000 and 1×10^{-6}, respectively. We fitted LCA models with 2 to 10 latent classes to determine the optimal number of classes, using criteria including the Akaike Information Criterion (AIC), Bayesian Information Criterion (BIC), likelihood ratio statistic (G^2), and mean posterior probability. Lower values of AIC, BIC, and G^2 indicate better model fit, while a mean posterior probability greater than 0.70 suggests classification with acceptable uncertainty. Our study showed that the AIC, BIC, and G^2 statistics stabilized after three latent classes (Figure S1). Table S1 presented the mean posterior probabilities, prevalence of latent classes, and item-response probabilities for models with 1 to 4 latent classes. In the three-latent-class solution, all mean posterior probabilities were ≥ 0.77. In contrast, the mean posterior probabilities for latent classes 2 and 4 in the four-latent-class solution were 0.67 and 0.69, both below 0.70. Therefore, the three-latent-class solution was considered optimal, representing high, medium and low SES, respectively.

Figure S2. AIC, BIC, and G^2 statistics in models with different numbers of latent classes in the UK biobank

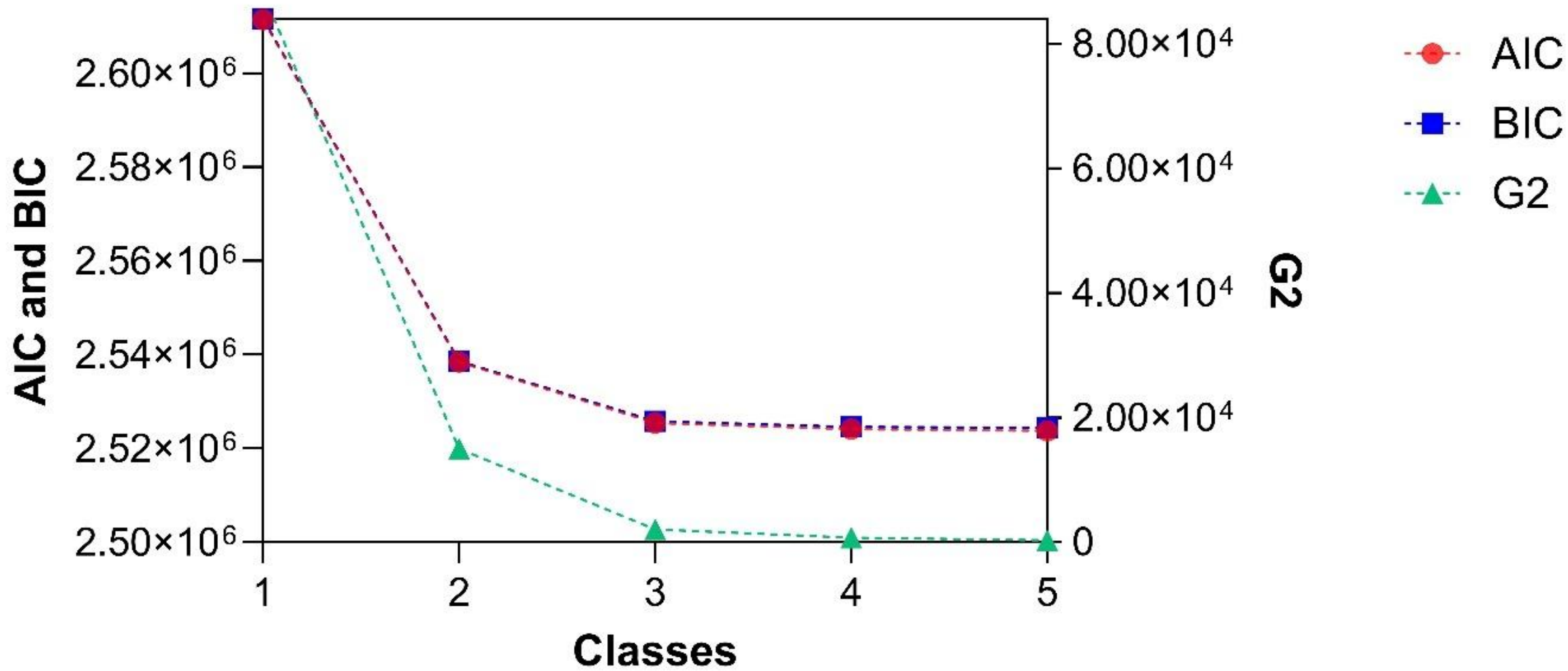

Note: Lower values of AIC, BIC, and G^2 indicate better model fit.

Abbreviations: AIC, Akaike information criterion; BIC, Bayesian information criterion; G^2, likelihood ratio statistic.

Figure S3. Directed acyclic graph for the hypothesized causal relationships among socioeconomic status, healthy lifestyle, and incident inflammatory bowel disease

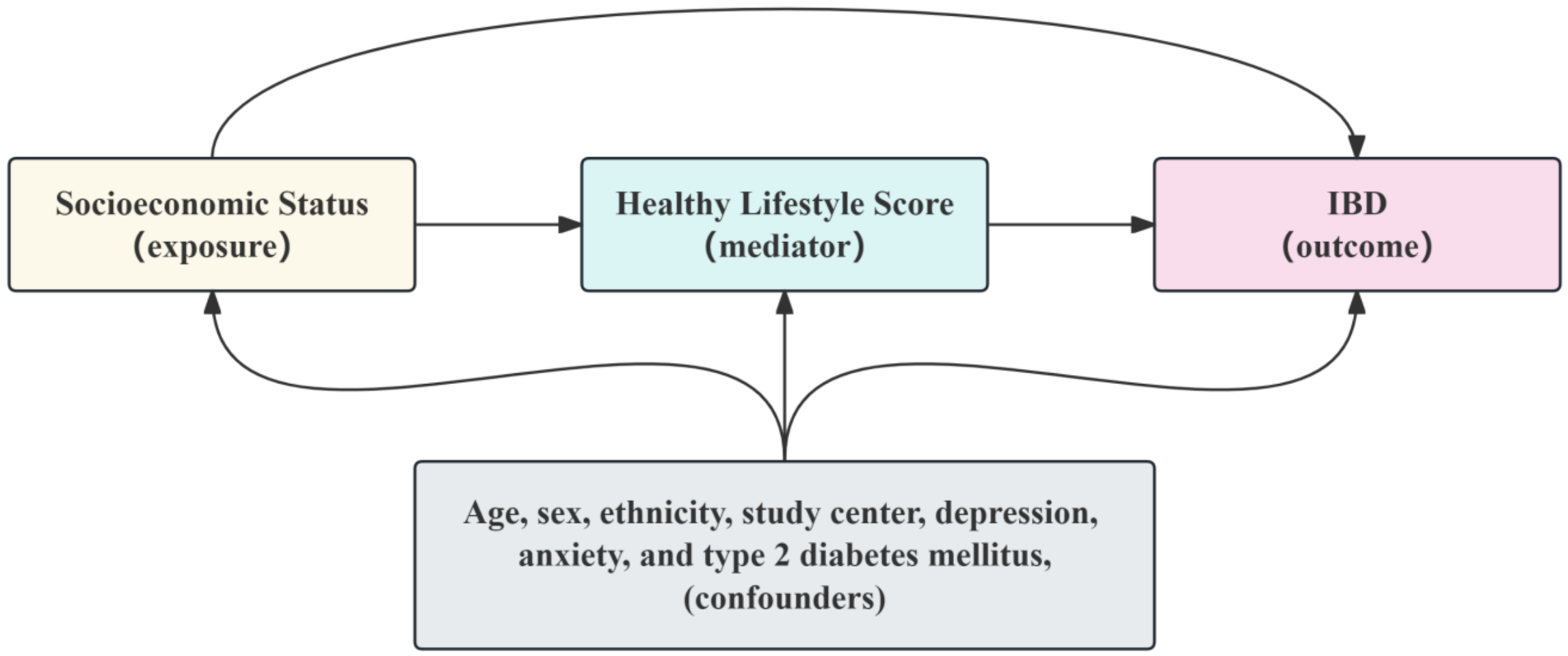

Note: The directed acyclic graph illustrates the assumed causal structure underlying the mediation analysis. Socioeconomic status (SES) was considered the exposure, healthy lifestyle score the proposed mediator, and incident IBD (including ulcerative colitis and Crohn's disease) the outcome. SES was operationalized as a composite of three dimensions: household income, educational attainment, and employment status. The healthy lifestyle score was constructed from six components: never smoking, moderate alcohol drinking, healthy diet, regular physical activity, normal body mass index, and healthy sleep duration. Baseline covariates, including age, sex, ethnicity, study center, depression, anxiety, and type 2 diabetes mellitus, were treated as potential confounders of the exposure–outcome, exposure–mediator, and mediator–outcome associations.

Table S1. Adherence to JoGH's GRABDROP guidelines items

**JoGH guideline items**
1. Please list all papers published by each co-author in previous 3 years that were based on secondary analysis of a big data repository

[1] Y. Chen, Q. Zhang, S. Liu, S. Zhu, J. Wu, S. Zhang, P. Li, S. Wu, Bidirectional association of rheumatoid arthritis and inflammatory bowel diseases: a large-scale prospective cohort study, Rheumatology 64 (2025) 5716-5725, https://doi.org/10.1093/rheumatology/keaf337.

[2] Y. Li, S. Liu, Q. Zhang, S. Zhang, S. Wu, Dietary index for gut microbiota and risk of incident irritable bowel syndrome: a large-scale prospective cohort study, Nutr. J. 24 (2025) 157, https://doi.org/10.1186/s12937-025-01224-3.

[3] S. Liang, Y. Zhou, Q. Zhang, S. Yu, S. Wu, Ultra-processed foods and risk of all-cause mortality: an updated systematic review and dose-response meta-analysis of prospective cohort studies, Syst. Rev. 14 (2025) 53, https://doi.org/10.1186/s13643-025-02800-8.

[4] H. Liu, S. Liu, Q. Zhang, S. Zhang, S. Zhu, S. Wu, Long-term risk of irritable bowel syndrome associated with triglyceride-glucose related indices: a large-scale prospective cohort study, Ther. Adv. Gastroenterol. 18 (2025) 1128566772, https://doi.org/10.1177/17562848251383796.

[5] H. Song, Y. Zhou, S. Liu, Q. Zhang, S. Zhang, S. Zhu, S. Wu, Long-term risk of inflammatory bowel disease in patients with irritable bowel syndrome: the cross-sectional and longitudinal relationship, J. Gastroenterol. 61 (2026) 139-149, https://doi.org/10.1007/s00535-025-02304-1.

[6] S. Wu, S. Xie, C. Yuan, Z. Yang, S. Liu, Q. Zhang, F. Sun, J. Wu, S. Zhan, S. Zhu, S. Zhang, Inflammatory bowel disease and long-term risk of cancer: a prospective cohort study among half a million adults in uk biobank, Inflamm. Bowel Dis. 29 (2023) 384-395, https://doi.org/10.1093/ibd/izac096.

[7] S. Wu, Z. Yang, S. Liu, Q. Zhang, S. Zhang, S. Zhu, Ultra-processed food consumption and long-term risk of irritable bowel syndrome: a large-scale prospective cohort study, Clin. Gastroenterol. Hepatol. 22 (2024) 1497-1507, https://doi.org/10.1016/j.cgh.2024.01.040.

[8] S. Wu, Z. Yang, S. Liu, Q. Zhang, S. Zhang, S. Zhu, Frailty status and risk of irritable bowel syndrome in middle-aged and older adults: a large-scale prospective cohort?study, Eclinicalmedicine 56 (2022) 101807, https://doi.org/10.1016/j.eclinm.2022.101807.

[9] S. Wu, Z. Yang, C. Yuan, S. Liu, Q. Zhang, S. Zhang, S. Zhu, Coffee and tea intake with long-term risk of irritable bowel syndrome: a large-scale prospective cohort study, Int. J. Epidemiol. 52 (2023) 1459-1472, https://doi.org/10.1093/ije/dyad024.

[10] S. Wu, C. Yuan, Z. Yang, S. Liu, Q. Zhang, S. Zhang, S. Zhu, Non-alcoholic fatty liver is associated with increased risk of irritable bowel syndrome: a prospective cohort study, Bmc Med. 20 (2022) 262, https://doi.org/10.1186/s12916-022-02460-8.

[11] S. Wu, C. Yuan, S. Liu, Q. Zhang, Z. Yang, F. Sun, S. Zhan, S. Zhu, S. Zhang, Irritable bowel syndrome and long-term risk of cancer: a prospective cohort study among 0.5 million adults in uk biobank., The American Journal of Gastroenterology 117 (2022) 785-793, https://doi.org/10.14309/ajg.0000000000001674.

[12] S. Yu, Y. Zhou, S. Liu, Q. Zhang, S. Zhang, S. Zhu, S. Wu, Both general and central obesity are associated with increased risk of irritable bowel syndrome: a large-scale prospective cohort study, The American Journal of Clinical Nutrition 121 (2025) 1054-1062, https://doi.org/10.1016/j.ajcnut.2025.03.001.

[13] Q. Zhang, P. Gui, S. Liu, S. Zhu, S. Zhang, S. Wu, Long-term risk of inflammatory bowel disease with sarcopenia status: a large-scale prospective cohort study, Geriatr. Gerontol. Int. 26 (2026) e70475, https://doi.org/10.1111/ggi.70475.

[14] Q. Zhang, S. Liu, J. Wu, S. Zhu, Y. Wu, S. Wu, S. Zhang, Non-alcoholic fatty liver degree and long-term risk of incident inflammatory bowel disease: a large-scale prospective cohort study, Chin. Med. J. 137 (2024) 1705-1714, https://doi.org/10.1097/CM9.0000000000002859.

[15] Q. Zhang, S. Liu, C. Yuan, F. Sun, S. Zhu, S. Guo, S. Wu, S. Zhang, Frailty and pre-frailty with long-term risk of elderly-onset inflammatory bowel disease: a large-scale prospective cohort study, Ann. Epidemiol. 88 (2023) 30-36, https://doi.org/10.1016/j.annepidem.2023.10.006.

[16] Q. Zhang, S. Liu, S. Zhu, A. Li, X. Sun, S. Wu, S. Zhang, Serum vitamin d levels and long-term risk of elderly-onset inflammatory bowel disease: a large-scale prospective cohort study, The American Journal of Medicine 138 (2025) 1374-1383, https://doi.org/10.1016/j.amjmed.2025.05.017.

[17] Q. Zhang, S. Liu, S. Zhu, J. Wu, S. Zhang, S. Wu, Association of healthy lifestyle, metabolic alterations and lower mortality risk of ibd patients: a prospective cohort and mediation analysis, Fam. Med. Community Health 13 (2025) e3514, https://doi.org/10.1136/fmch-2025-003514.

[18] Q. Zhang, Y. Wang, S. Liu, S. Zhu, P. Li, S. Wu, Mortality risk associated with masld, masld type and different cardiometabolic risk factors in ibd patients: a long-term prospective cohort study, Dig. Liver Dis. 57 (2025) 744-752, https://doi.org/10.1016/j.dld.2024.11.002.

[19] Q. Zhang, Y. Wang, S. Liu, S. Zhu, P. Li, S. Zhang, Z. Yang, S. Wu, Mortality risk associated with general and central obesity in inflammatory bowel disease patients: a long-term prospective cohort study, International Journal of Obesity (2005) 49 (2025) 2303-2310, https://doi.org/10.1038/s41366-025-01879-2.

[20] Q. Zhang, F. Xu, S. Liu, S. Zhu, S. Zhang, J. Wu, S. Wu, Long-term risk of cardiovascular disease associated withmasld and different cardiometabolic risk factors inibd patients: a prospective cohort study, Liver Int. 44 (2024) 2315-2328, https://doi.org/10.1111/liv.15999.

[21] Q. Zhang, F. Xu, Z. Wang, S. Liu, S. Zhu, S. Zhang, S. Wu, Long-term risk of inflammatory bowel disease with masld: a large-scale prospective cohort study in uk biobank, J. Gastroenterol. Hepatol. 40 (2025) 855-865, https://doi.org/10.1111/jgh.16880.

[22] Q. Zhang, F. Xu, S. Liu, S. Zhu, S. Zhang, S. Wu, W. Li, Long-term risk of bowel resection and mortality associated with sleep behaviours and sleep pattern in patients with inflammatory bowel disease: a large-scale prospective cohort study., Fam. Med. Community Health 14 (2026), https://doi.org/10.1136/fmch-2025-003787.

[23] Y. Zhou, S. Liu, Q. Zhang, S. Zhang, S. Wu, S. Zhu, Bidirectional association between type 2 diabetes and irritable bowel syndrome: a large-scale prospective cohort study, Diabetes, Obesity and Metabolism 26 (2024) 5107-5115, https://doi.org/10.1111/dom.15852.

[24] Y. Zhou, Z. Yang, S. Liu, S. Xie, Q. Zhang, S. Zhang, S. Zhu, S. Wu, Long-term risk of irritable bowel syndrome associated with masld, masld type and different cardiometabolic risk factors: a large-scale prospective cohort study, Bmc Gastroenterol. 25 (2025) 576, https://doi.org/10.1186/s12876-025-04165-7.

2. Please explain the key elements of your study design and the use of the available datasets that make your study an original scientific contribution
This study used the UK Biobank (UKB), a large-scale prospective cohort of over 500,000 adults aged 37–73 years recruited from 22 centres across England, Wales, and Scotland (2006–2010), with a median follow-up of 14.57 years. Incident IBD (UC and CD) was ascertained through linkage to primary care and hospital inpatient data. The original scientific contribution of this study is threefold: (1) it is the first large-scale prospective cohort study to comprehensively examine the association between a composite SES measure — derived from household income, education level, and employment status using latent profile analysis — and long-term risk of incident IBD; (2) it formally quantifies the mediating role of a multi-component healthy lifestyle score in the SES–IBD pathway using formal causal mediation analysis; and (3) it evaluates the joint effects of SES and lifestyle on IBD risk, providing actionable targets for reducing socioeconomic health disparities in IBD prevention.
3. Please list all publications that addressed similar research questions in the same dataset and indicate where you cited them in your paper
No prior publication has examined the association between composite SES and incident IBD risk with mediation analysis in the UK Biobank. Reference (Ye et al., 2023) used UK Biobank to examine SES–infectious disease associations mediated by lifestyle factors, and is cited in the Introduction (paragraph 3). Reference (Sun et al., 2023) used UK Biobank to investigate lifestyle factors in IBD development, and is cited in the Methods section for lifestyle score construction and covariate selection.<br><br>*Ye X, Wang Y, Zou Y, Tu J, Tang W, Yu R, et al. Associations of socioeconomic status with infectious diseases mediated by lifestyle, environmental pollution and chronic comorbidities: a comprehensive evaluation based on UK Biobank. Infect Dis Poverty 2023;12:5.*<br>*Sun Y, Yuan S, Chen X, Sun J, Kalla R, Yu L, et al. The Contribution of Genetic Risk and Lifestyle Factors in the Development of Adult-Onset Inflammatory Bowel Disease: A Prospective Cohort Study. The American journal of gastroenterology 2023;118:511-522.*
4. Please explain how you addressed multiple testing through an appropriately rigorous statistical threshold and indicate this in the methods section
Subgroup analyses by age and sex were pre-specified to assess the consistency and robustness of the main associations, rather than for hypothesis generation. Effect modification was formally evaluated using multiplicative interaction terms in the fully adjusted model (Model 2).

Given the pre-specified and confirmatory nature of these analyses, no formal correction for multiple testing was applied; however, all subgroup and interaction results should be interpreted cautiously, as the risk of type I error may be increased.

5. Please declare to what extent have AI chatbots been used in developing your paper and to which parts of the paper did they contribute
No AI chatbots (ChatGPT or similar) were used in the conceptualization, study design, data analysis, interpretation of results, or drafting of this manuscript.

Table S2. STROBE Statement-Checklist of items that should be included in reports of *cohort studies*

	Item No	Recommendation	Page No
**Title and abstract**	1	(*a*) Indicate the study's design with a commonly used term in the title or the abstract	1
		(*b*) Provide in the abstract an informative and balanced summary of what was done and what was found	2
**Introduction**			
Background/rationale	2	Explain the scientific background and rationale for the investigation being reported	3
Objectives	3	State specific objectives, including any prespecified hypotheses	3
**Methods**			
Study design	4	Present key elements of study design early in the paper	4
Setting	5	Describe the setting, locations, and relevant dates, including periods of recruitment, exposure, follow-up, and data collection	4
Participants	6	(*a*) Give the eligibility criteria, and the sources and methods of selection of participants. Describe methods of follow-up	4
		(*b*) For matched studies, give matching criteria and number of exposed and unexposed	N/A
Variables	7	Clearly define all outcomes, exposures, predictors, potential confounders, and effect modifiers. Give diagnostic criteria, if applicable	4,5
Data sources/ measurement	8*	For each variable of interest, give sources of data and details of methods of assessment (measurement). Describe comparability of assessment methods if there is more than one group	4,5
Bias	9	Describe any efforts to address potential sources of bias	5
Study size	10	Explain how the study size was arrived at	4
Quantitative variables	11	Explain how quantitative variables were handled in the analyses. If applicable, describe which groupings were chosen and why	4,5
Statistical methods	12	(*a*) Describe all statistical methods, including those used to control for confounding	5,6
		(*b*) Describe any methods used to examine subgroups and interactions	5,6
		(*c*) Explain how missing data were addressed	5,6
		(*d*) If applicable, explain how loss to follow-up was addressed	5,6
		(*e*) Describe any sensitivity analyses	6

**Results**			
Participants	13*	(a) Report numbers of individuals at each stage of study—eg numbers potentially eligible, examined for eligibility, confirmed eligible, included in the study, completing follow-up, and analysed	7
		(b) Give reasons for non-participation at each stage	7
		(c) Consider use of a flow diagram	7
Descriptive data	14*	(a) Give characteristics of study participants (eg demographic, clinical, social) and information on exposures and potential confounders	7
		(b) Indicate number of participants with missing data for each variable of interest	7
		(c) Summarise follow-up time (eg, average and total amount)	7
Outcome data	15*	Report numbers of outcome events or summary measures over time	7,8

Table S3. Mean posterior probabilities, prevalence of latent classes, and item-response probabilities in models with three to four latent classes

	Item		Latent class 1	Latent class 2	Latent class 3	Latent class 4
**Three-latent-class solution**		Mean posterior probabilities	0.80	0.77	0.93	-
		Prevalence	0.20	0.53	0.27	-
	Income (£)	< 18,000	0.00	0.00	**0.72**	-
		18,000–30,999	0.00	0.40	0.22	-
		31,000–51,999	0.23	**0.43**	0.04	-
		52,000–100,000	**0.54**	0.17	0.01	-
		> 100,000	0.23	0.00	0.01	-
	Education	College or University degree	**0.73**	**0.30**	0.15	-
		A levels/AS levels or equivalent	0.13	0.13	0.08	-
		O levels/GCSEs or equivalent	0.09	0.27	0.22	-
		CSEs or equivalent	0.01	0.07	0.06	-
		NVQ or HND or HNC or equivalent	0.02	0.08	0.08	-
		Other professional qualifications	0.03	0.06	0.05	-
		None of the above	0.00	0.08	**0.35**	-
	Employment	Employed	**0.96**	**0.98**	**0.84**	-
		Unemployed	0.04	0.02	0.16	-
**Four-latent-class solution**		Mean posterior probabilities	0.77	0.67	0.72	0.69
		Prevalence	0.20	0.49	0.09	0.22
	Income (£)	< 18,000	0.00	0.00	0.35	**0.72**
		18,000–30,999	0.00	0.36	**0.39**	0.17
		31,000–51,999	0.22	**0.44**	0.19	0.07
		52,000–100,000	**0.54**	0.20	0.06	0.03
		> 100,000	0.24	0.00	0.00	0.02
	Education	College or University degree	**0.75**	**0.34**	0.16	0.15
		A levels/AS levels or equivalent	0.12	0.16	0.05	0.10
		O levels/GCSEs or equivalent	0.08	0.28	0.21	0.22
		CSEs or equivalent	0.01	0.09	0.01	0.09

	NVQ or HND or HNC or equivalent	0.02	0.07	0.10	0.07
	Other professional qualifications	0.03	0.05	0.09	0.03
	None of the above	0.00	0.00	**0.37**	**0.34**
Employment	Employed	**0.96**	**0.97**	**1.00**	**0.75**
	Unemployed	0.04	0.03	0.00	0.25

Note: The maximal item-response probabilities for each latent class were marked in bold.

Latent class with mean posterior probability higher than 0.7 as classification with acceptable uncertainty.

Table S4. Subgroup analysis of each SES item with risk incident IBD, UC and CD by age

Age subgroup	No. of participants	Incident IBD		Incident UC		Incident CD	
		cases	HR (95%CI, P value)	cases	HR (95%CI, P value)	cases	HR (95%CI, P value)
**Age≥60 years**							
Household income							
High	18858	117	0.71(0.58-0.88, 0.001)	85	0.71(0.55-0.91, 0.006)	38	0.67(0.46-0.97, 0.032)
Medium	79713	549	0.82(0.72-0.93, 0.002)	414	0.85(0.73-0.99, 0.038)	170	0.72(0.58-0.90, 0.004)
Low	49157	402	Reference	288	Reference	144	Reference
Education level							
College or above	42805	246	0.65(0.55-0.77, <0.001)	175	0.63(0.51-0.76, <0.001)	84	0.65(0.48-0.86, 0.003)
High school or equivalent	69511	518	0.85(0.74-0.98, 0.026)	390	0.87(0.74-1.03, 0.108)	163	0.78(0.61-1.00, 0.051)
Less than high school	35412	304	Reference	222	Reference	105	Reference
Employment status							
Employed, student or retired	143622	1026	0.71(0.52-0.97, 0.029)	760	0.82(0.56-1.21, 0.327)	335	0.56(0.34-0.92, 0.021)
Unemployed	4106	42	Reference	27	Reference	17	Reference
**Age<60 years**							
Household income							
High	80988	373	0.62(0.53-0.74, <0.001)	258	0.64(0.52-0.78, <0.001)	133	0.55(0.42-0.72, <0.001)
Medium	113638	700	0.84(0.72-0.97, 0.018)	497	0.88(0.74-1.06, 0.176)	259	0.76(0.60-0.96, 0.019)

Low	32153	240	Reference	161	Reference	99	Reference
Education level							
College or above	90310	419	0.50(0.41-0.59, <0.001)	289	0.48(0.39-0.59, <0.001)	157	0.46(0.35-0.62, <0.001)
High school or equivalent	118182	722	0.65(0.55-0.77, <0.001)	505	0.64(0.53-0.79, <0.001)	266	0.59(0.45-0.77, <0.001)
Less than high school	18287	172	Reference	122	Reference	68	Reference
Employment status							
Employed, student or retired	205232	1139	0.69(0.59-0.81, <0.001)	794	0.68(0.56-0.82, <0.001)	424	0.71(0.54-0.92, 0.010)
Unemployed	21547	174	Reference	122	Reference	67	Reference

Note: IBD: inflammatory bowel disease; UC: ulcerative colitis; CD: Crohn's disease. For each item of socioeconomic status, all HRs were adjusted for age, sex, ethnicity, study center, depression, anxiety, type 2 diabetes and the other two items of socioeconomic factors. P for interaction for household income was 0.183, 0.106 and 0.711 for IBD, UC and CD, respectively. P for interaction for education level was for 0.001, <0.001 and 0.112 for IBD, UC and CD, respectively. P for interaction for employment status was 0.001, <0.001 and 0.179 for IBD, UC and CD, respectively.

Table S5. Subgroup analysis of each SES item with risk incident IBD, UC and CD by sex

**Sex subgroup**	**No. of participants**	**Incident IBD**		**Incident UC**		**Incident CD**	
		**cases**	**HR (95%CI, P value)**	**cases**	**HR (95%CI, P value)**	**cases**	**HR (95%CI, P value)**
**Male**							
Household income							
High	52928	295	0.68(0.57-0.81, <0.001)	205	0.65(0.53-0.79, <0.001)	103	0.69(0.51-0.92, 0.010)
Medium	93173	671	0.87(0.75-1.00, 0.044)	507	0.90(0.76-1.06, 0.190)	206	0.77(0.61-0.99, 0.040)
Low	35694	295	Reference	216	Reference	101	Reference
Education level							
College or above	65860	367	0.58(0.50-0.69, <0.001)	262	0.55(0.45-0.66, <0.001)	121	0.60(0.45-0.81, <0.001)
High school or equivalent	88435	634	0.76(0.65-0.88, <0.001)	467	0.73(0.62-0.87, <0.001)	207	0.77(0.59-1.01, 0.056)
Less than high school	27500	260	Reference	199	Reference	82	Reference
Employment status							

Employed, student or retired	170955	1150	0.63(0.52-0.77, <0.001)	849	0.65(0.51-0.82, <0.001)	372	0.62(0.44-0.87, 0.006)
Unemployed	10840	111	Reference	79	Reference	38	Reference
**Female**							
Household income							
High	46918	195	0.59(0.49-0.71, <0.001)	138	0.63(0.50-0.79, <0.001)	68	0.46(0.34-0.62, <0.001)
Medium	100178	578	0.78(0.68-0.90, <0.001)	404	0.82(0.70-0.97, 0.022)	223	0.71(0.57-0.88, 0.002)
Low	45616	347	Reference	233	Reference	142	Reference
Education level							
College or above	67255	298	0.58(0.48-0.69, <0.001)	202	0.59(0.47-0.74, <0.001)	120	0.51(0.38-0.68, <0.001)
High school or equivalent	99258	606	0.78(0.66-0.91, 0.002)	428	0.83(0.68-1.01, 0.057)	222	0.63(0.49-0.81, <0.001)
Less than high school	26199	216	Reference	145	Reference	91	Reference
Employment status							
Employed, student or retired	177899	1015	0.78(0.63-0.96, 0.017)	705	0.80(0.62-1.03, 0.079)	387	0.72(0.53-0.99, 0.040)
Unemployed	14813	105	Reference	70	Reference	46	Reference

Note: IBD: inflammatory bowel disease; UC: ulcerative colitis; CD: Crohn's disease. For each item of socioeconomic status, all HRs were adjusted for age, sex, ethnicity, study center, depression, anxiety, type 2 diabetes and the other two items of socioeconomic factors. P for interaction for household income was 0.149, 0.120 and 0.220 for IBD, UC and CD, respectively. P for interaction for education level was for 0.849, 0.754 and 0.578 for IBD, UC and CD, respectively. P for interaction for employment status was 0.184, 0.263 and 0.628 for IBD, UC and CD, respectively.

Table S6. Joint associations of SES pattern and healthy lifestyle score with incident IBD, UC and CD

**SES pattern and healthy lifestyle score**	**No. of participants**	**Incident IBD**		**Incident UC**		**Incident CD**	
		**cases**	**HR (95%CI, P value)**	**cases**	**HR (95%CI, P value)**	**cases**	**HR (95%CI, P value)**
Healthy lifestyle score 5-6							
High SES	28100	114	0.37(0.29-0.46, <0.001)	76	0.37(0.28-0.48, <0.001)	43	0.31(0.22-0.45, <0.001)
Medium SES	59846	268	0.40(0.33-0.48, <0.001)	189	0.42(0.34-0.52, <0.001)	93	0.32(0.24-0.43, <0.001)

Low SES	23885	126	0.46(0.37-0.58, <0.001)	91	0.49(0.38-0.64, <0.001)	42	0.36(0.25-0.52, <0.001)
Healthy lifestyle score 3-4							
High SES	39361	160	0.36(0.30-0.44, <0.001)	115	0.39(0.30-0.49, <0.001)	54	0.28(0.20-0.39, <0.001)
Medium SES	107504	713	0.58(0.50-0.68, <0.001)	524	0.63(0.53-0.76, <0.001)	238	0.46(0.36-0.58, <0.001)
Low SES	56462	441	0.68(0.58-0.80, <0.001)	313	0.71(0.59-0.86, <0.001)	162	0.59(0.46-0.76, <0.001)
Healthy lifestyle score 0-2							
High SES	8831	55	0.55(0.41-0.73, <0.001)	34	0.50(0.34-0.72, <0.001)	23	0.54(0.34-0.85, 0.008)
Medium SES	29780	269	0.79(0.66-0.94, 0.009)	201	0.87(0.70-1.07, 0.184)	90	0.63(0.47-0.84, 0.002)
Low SES	20738	235	Reference	160	Reference	98	Reference

Note: SES: socioeconomic status; IBD: inflammatory bowel disease; UC: ulcerative colitis; CD: Crohn's disease. All HRs were adjusted for age, sex, ethnicity, study center, depression, anxiety and type 2 diabetes. The healthy lifestyle score consisted of never smoking, moderate alcohol drinking, healthy diet, regular physical activity, normal BMI, and healthy sleep duration.

Table S7. Joint associations of SES score and healthy lifestyle score with incident IBD, UC and CD

SES score and healthy lifestyle score	No. of participants	Incident IBD		Incident UC		Incident CD	
		cases	HR (95%CI, P value)	cases	HR (95%CI, P value)	cases	HR (95%CI, P value)
Healthy lifestyle score 5-6							
SES 5-6	54045	235	0.32(0.26-0.39, <0.001)	157	0.32(0.25-0.41, <0.001)	89	0.28(0.20-0.38, <0.001)
SES score 3-4	49612	226	0.33(0.27-0.41, <0.001)	160	0.35(0.27-0.45, <0.001)	77	0.26(0.18-0.36, <0.001)
SES score 0-2	8174	47	0.41(0.30-0.57, <0.001)	39	0.51(0.35-0.74, <0.001)	12	0.24(0.13-0.45, <0.001)
Healthy lifestyle score 3-4							
SES score 5-6	80542	420	0.38(0.31-0.45, <0.001)	311	0.42(0.33-0.52, <0.001)	132	0.27(0.20-0.37, <0.001)
SES score 3-4	98521	702	0.51(0.43-0.61, <0.001)	505	0.55(0.44-0.67, <0.001)	251	0.42(0.32-0.56, <0.001)
SES score 0-2	24264	192	0.56(0.45-0.69, <0.001)	136	0.59(0.46-0.77, <0.001)	71	0.48(0.34-0.68, <0.001)
Healthy lifestyle score							

0-2							
SES score 5-6	18755	138	0.53(0.42-0.67, <0.001)	91	0.52(0.39-0.68, <0.001)	55	0.50(0.34-0.71, <0.001)
SES score 3-4	29503	268	0.65(0.53-0.79, <0.001)	201	0.72(0.57-0.91, 0.007)	91	0.52(0.38-0.72, <0.001)
SES score 0-2	11091	153	Reference	103	Reference	65	Reference

Note: SES: socioeconomic status; IBD: inflammatory bowel disease; UC: ulcerative colitis; CD: Crohn's disease. All HRs were adjusted for age, sex, ethnicity, study center, depression, anxiety and type 2 diabetes. The healthy lifestyle score consisted of never smoking, moderate alcohol drinking, healthy diet, regular physical activity, normal BMI, and healthy sleep duration.

Table S8. Subgroup analysis for joint associations of SES pattern and healthy lifestyle score with incident IBD, UC and CD by age

**Age subgroup**	**No. of participants**	**Incident IBD**		**Incident UC**		**Incident CD**	
		**cases**	**HR (95%CI, P value)**	**cases**	**HR (95%CI, P value)**	**cases**	**HR (95%CI, P value)**
**Age≥60 years**							
Healthy lifestyle score 5-6							
High SES	4396	28	0.56(0.37-0.84, 0.005)	18	0.52(0.31-0.87, 0.012)	11	0.56(0.29-1.08, 0.085)
Medium SES	22633	108	0.43(0.33-0.55, <0.001)	82	0.47(0.35-0.64, <0.001)	32	0.32(0.20-0.50, <0.001)
Low SES	14460	81	0.51(0.38-0.67, <0.001)	58	0.54(0.38-0.75, <0.001)	27	0.41(0.26-0.67, <0.001)
Healthy lifestyle score 3-4							
High SES	7612	45	0.51(0.36-0.72, <0.001)	36	0.59(0.40-0.88, 0.009)	13	0.38(0.21-0.71, 0.002)
Medium SES	40826	287	0.62(0.50-0.77, <0.001)	216	0.68(0.53-0.88, 0.003)	89	0.49(0.35-0.70, <0.001)
Low SES	33675	282	0.76(0.62-0.94, 0.012)	205	0.81(0.63-1.05, 0.114)	98	0.66(0.47-0.93, 0.018)
Healthy lifestyle score 0-2							
High SES	1801	10	0.48(0.25-0.92, 0.026)	5	0.35(0.14-0.85, 0.021)	5	0.63(0.25-1.58, 0.323)
Medium SES	10758	124	0.85(0.66-1.11, 0.240)	82	0.98(0.73-1.34, 0.922)	28	0.60(0.38-0.95, 0.031)
Low SES	11567	103	Reference	85	Reference	49	Reference
**Age < 60 years**							
Healthy lifestyle score							

5-6							
High SES	23704	86	0.31(0.23-0.41, <0.001)	58	0.31(0.22-0.43, <0.001)	32	0.25(0.16-0.40, <0.001)
Medium SES	37213	160	0.36(0.28-0.47, <0.001)	107	0.36(0.27-0.49, <0.001)	61	0.31(0.21-0.45, <0.001)
Low SES	9425	45	0.40(0.29-0.57, <0.001)	33	0.44(0.29-0.67, <0.001)	15	0.30(0.17-0.53, <0.001)
Healthy lifestyle score 3-4							
High SES	31749	115	0.30(0.23-0.39, <0.001)	79	0.31(0.22-0.42, <0.001)	41	0.25(0.16-0.37, <0.001)
Medium SES	66678	426	0.53(0.43-0.66, <0.001)	308	0.57(0.44-0.74, <0.001)	64	0.42(0.30-0.58, <0.001)
Low SES	22787	159	0.58(0.46-0.74, <0.001)	108	0.59(0.44-0.79, <0.001)	149	0.52(0.36-0.76, <0.001)
Healthy lifestyle score 0-2							
High SES	7030	45	0.53(0.37-0.75, <0.001)	29	0.50(0.32-0.77, 0.002)	18	0.49(0.28-0.84, 0.010)
Medium SES	19022	166	0.72(0.56-0.92, 0.008)	119	0.76(0.57-1.01, 0.062)	62	0.62(0.42-0.90, 0.013)
Low SES	9171	111	Reference	75	Reference	49	Reference

Note: SES: socioeconomic status; IBD: inflammatory bowel disease; UC: ulcerative colitis; CD: Crohn's disease. All HRs were adjusted for age, sex, ethnicity, study center, depression, anxiety and type 2 diabetes. The healthy lifestyle score consisted of never smoking, moderate alcohol drinking, healthy diet, regular physical activity, normal BMI, and healthy sleep duration. P for interaction was 0.010, 0.003 and 0.440 for IBD, UC and CD, respectively.

Table S9. Subgroup analysis for joint associations of SES pattern and healthy lifestyle score with incident IBD, UC and CD by sex

**Sex subgroup**	**No. of participants**	**Incident IBD**		**Incident UC**		**Incident CD**	
		**cases**	**HR (95%CI, P value)**	**cases**	**HR (95%CI, P value)**	**cases**	**HR (95%CI, P value)**
**Male**							
Healthy lifestyle score 5-6							
High SES	12377	54	0.41(0.30-0.57, <0.001)	32	0.33(0.22-0.50, <0.001)	23	0.56(0.33-0.95, 0.030)
Medium SES	23505	121	0.48(0.37-0.62, <0.001)	89	0.48(0.36-0.64, <0.001)	37	0.47(0.30-0.74, 0.001)
Low SES	7712	42	0.50(0.35-0.71, <0.001)	28	0.45(0.29-0.68, <0.001)	16	0.62(0.35-1.11, 0.109)
Healthy lifestyle score 3-4							

High SES	21373	106	0.47(0.36-0.61, <0.001)	78	0.47(0.35-0.64, <0.001)	35	0.49(0.31-0.78, 0.003)
Medium SES	54740	389	0.67(0.55-0.82, <0.001)	296	0.69(0.54-0.87, 0.002)	117	0.64(0.45-0.92, 0.017)
Low SES	25113	207	0.77(0.62-0.97, 0.023)	155	0.78(0.60-1.01, 0.058)	70	0.84(0.57-1.24, 0.391)
Healthy lifestyle score 0-2							
High SES	5792	34	0.56(0.38-0.82, 0.003)	21	0.47(0.29-0.75, 0.002)	14	0.73(0.40-1.35, 0.316)
Medium SES	18721	181	0.92(0.73-1.16, 0.494)	135	0.93(0.71-1.22, 0.604)	58	0.94(0.63-1.41, 0.768)
Low SES	12462	127	Reference	94	Reference	40	Reference
**Female**							
Healthy lifestyle score 5-6							
High SES	15723	60	0.31(0.23-0.43, <0.001)	44	0.38(0.26-0.56, <0.001)	20	0.18(0.11-0.30, <0.001)
Medium SES	36341	147	0.32(0.25-0.41, <0.001)	100	0.36(0.26-0.49, <0.001)	56	0.22(0.15-0.32, <0.001)
Low SES	16173	84	0.40(0.30-0.53, <0.001)	63	0.48(0.34-0.68, <0.001)	26	0.23(0.15-0.37, <0.001)
Healthy lifestyle score 3-4							
High SES	17988	54	0.24(0.17-0.34, <0.001)	37	0.27(0.18-0.41, <0.001)	19	0.15(0.09-0.25, <0.001)
Medium SES	52764	324	0.48(0.39-0.60, <0.001)	228	0.56(0.42-0.73, <0.001)	121	0.33(0.24-0.45, <0.001)
Low SES	31349	234	0.57(0.45-0.71, <0.001)	158	0.62(0.47-0.83, 0.001)	92	0.42(0.30-0.59, <0.001)
Healthy lifestyle score 0-2							
High SES	3039	21	0.56(0.35-0.89, 0.015)	13	0.57(0.31-1.04, 0.066)	9	0.41(0.20-0.84, 0.014)
Medium SES	11059	88	0.62(0.47-0.83, 0.001)	66	0.77(0.55-1.09, 0.138)	32	0.41(0.26-0.63, <0.001)
Low SES	8276	108	Reference	66	Reference	58	Reference

Note: SES: socioeconomic status; IBD: inflammatory bowel disease; UC: ulcerative colitis; CD: Crohn's disease. All HRs were adjusted for age, sex, ethnicity, study center, depression, anxiety and type 2 diabetes. The healthy lifestyle score consisted of never smoking, moderate alcohol drinking, healthy diet, regular physical activity, normal BMI, and healthy sleep duration. P for interaction was 0.024, 0.270 and 0.014 for IBD, UC and CD, respectively.

Table S10. Subgroup analysis for joint associations of SES score and healthy lifestyle score with incident IBD, UC and CD by age

Age subgroup	No. of participants	Incident IBD		Incident UC		Incident CD	
		cases	HR (95%CI, P value)	cases	HR (95%CI, P value)	cases	HR (95%CI, P value)
**Age≥60 years**							
Healthy lifestyle score 5-6							
SES score 5-6	14539	72	0.35(0.25-0.48, <0.001)	49	0.34(0.23-0.50, <0.001)	26	0.34(0.20-0.57, <0.001)
SES score 3-4	22387	116	0.37(0.27-0.49, <0.001)	86	0.40(0.28-0.56, <0.001)	37	0.31(0.19-0.51, <0.001)
SES score 0-2	4563	29	0.46(0.30-0.70, <0.001)	23	0.53(0.32-0.87, 0.012)	7	0.28(0.12-0.65, 0.003)
Healthy lifestyle score 3-4							
SES score 5-6	23300	143	0.43(0.32-0.57, <0.001)	110	0.47(0.34-0.66, <0.001)	40	0.32(0.20-0.53, <0.001)
SES score 3-4	45505	363	0.57(0.44-0.73, <0.001)	268	0.60(0.45-0.81, <0.001)	122	0.51(0.33-0.76, 0.001)
SES score 0-2	13308	108	0.59(0.44-0.79, <0.001)	79	0.63(0.44-0.89, 0.009)	38	0.54(0.33-0.89, 0.015)
Healthy lifestyle score 0-2							
SES score 5-6	5347	43	0.56(0.39-0.82, 0.003)	28	0.52(0.33-0.83, 0.006)	16	0.57(0.31-1.06, 0.076)
SES score 3-4	13164	119	0.65(0.49-0.87, 0.003)	92	0.72(0.51-1.01, 0.059)	38	0.56(0.34-0.91, 0.020)
SES score 0-2	5615	75	Reference	52	Reference	28	Reference
**Age < 60 years**							
Healthy lifestyle score 5-6							
SES score 5-6	39506	163	0.30(0.22-0.39, <0.001)	108	0.30(0.21-0.42, <0.001)	63	0.24(0.16-0.36, <0.001)
SES score 3-4	27225	110	0.29(0.22-0.39, <0.001)	74	0.30(0.21-0.43, <0.001)	40	0.22(0.14-0.34, <0.001)
SES score 0-2	3611	18	0.36(0.21-0.60, <0.001)	16	0.49(0.28-0.86, 0.013)	5	0.20(0.08-0.52, <0.001)
Healthy lifestyle score 3-4							
SES score 5-6	57242	277	0.34(0.26-0.44, <0.001)	201	0.38(0.28-0.51, <0.001)	92	0.24(0.16-0.36, <0.001)
SES score 3-4	53016	339	0.45(0.35-0.58, <0.001)	237	0.48(0.36-0.66, <0.001)	129	0.36(0.25-0.53, <0.001)

SES score 0-2	10956	84	0.54(0.40-0.74, <0.001)	57	0.56(0.39-0.82, 0.003)	33	0.44(0.27-0.70, <0.001)
Healthy lifestyle score 0-2							
SES score 5-6	13408	95	0.50(0.37-0.67, <0.001)	63	0.50(0.34-0.72, <0.001)	39	0.44(0.28-0.70, <0.001)
SES score 3-4	16339	149	0.64(0.48-0.84, 0.001)	109	0.71(0.51-0.99, 0.043)	53	0.49(0.32-0.75, 0.001)
SES score 0-2	5476	78	Reference	51	Reference	37	Reference

Note: SES: socioeconomic status; IBD: inflammatory bowel disease; UC: ulcerative colitis; CD: Crohn's disease. All HRs were adjusted for age, sex, ethnicity, study center, depression, anxiety and type 2 diabetes. The healthy lifestyle score consisted of never smoking, moderate alcohol drinking, healthy diet, regular physical activity, normal BMI, and healthy sleep duration. P for interaction was 0.010, 0.005 and 0.394 for IBD, UC and CD, respectively.

Table S11. Subgroup analysis for joint associations of SES score and healthy lifestyle score with incident IBD, UC and CD by sex

Sex subgroup	No. of participants	Incident IBD		Incident UC		Incident CD	
		cases	HR (95%CI, P value)	cases	HR (95%CI, P value)	cases	HR (95%CI, P value)
**Male**							
Healthy lifestyle score 5-6							
SES score 5-6	23984	116	0.36(0.27-0.48, <0.001)	77	0.33(0.24-0.47, <0.001)	42	0.40(0.25-0.64, <0.001)
SES score 3-4	16986	83	0.36(0.26-0.48, <0.001)	58	0.35(0.24-0.50, <0.001)	30	0.40(0.24-0.67, <0.001)
SES score 0-2	2624	18	0.50(0.30-0.83, 0.007)	14	0.54(0.30-0.96, 0.037)	4	0.34(0.12-0.98, 0.045)
Healthy lifestyle score 3-4							
SES score 5-6	43437	256	0.44(0.34-0.56, <0.001)	193	0.46(0.35-0.62, <0.001)	74	0.39(0.25-0.60, <0.001)
SES score 3-4	46649	345	0.55(0.43-0.69, <0.001)	259	0.57(0.43-0.76, <0.001)	114	0.55(0.37-0.83, 0.005)
SES score 0-2	11140	101	0.68(0.51-0.91, 0.009)	77	0.72(0.52-1.01, 0.058)	34	0.70(0.43-1.15, 0.159)
Healthy lifestyle score 0-2							
SES score 5-6	12139	94	0.58(0.43-0.78, <0.001)	61	0.53(0.37-0.76, <0.001)	38	0.71(0.44-1.16, 0.178)
SES score 3-4	17959	161	0.68(0.52-0.88, 0.003)	127	0.75(0.55-1.01, 0.061)	45	0.58(0.36-0.92, 0.021)
SES score 0-2	6877	87	Reference	62	Reference	29	Reference

**Female**							
Healthy lifestyle score 5-6							
SES score 5-6	30061	119	0.27(0.20-0.37, <0.001)	80	0.29(0.20-0.43, <0.001)	47	0.19(0.12-0.29, <0.001)
SES score 3-4	32626	143	0.28(0.21-0.38, <0.001)	102	0.32(0.23-0.47, <0.001)	47	0.17(0.11-0.27, <0.001)
SES score 0-2	5550	29	0.33(0.21-0.51, <0.001)	25	0.45(0.27-0.75, 0.002)	8	0.17(0.08-0.37, <0.001)
Healthy lifestyle score 3-4							
SES score 5-6	37105	164	0.30(0.22-0.40, <0.001)	118	0.34(0.24-0.49, <0.001)	58	0.19(0.12-0.29, <0.001)
SES score 3-4	51872	357	0.45(0.34-0.58, <0.001)	246	0.49(0.35-0.68, <0.001)	137	0.32(0.22-0.46, <0.001)
SES score 0-2	13124	91	0.44(0.32-0.60, <0.001)	59	0.45(0.30-0.67, <0.001)	37	0.33(0.21-0.52, <0.001)
Healthy lifestyle score 0-2							
SES score 5-6	6616	44	0.45(0.30-0.66, <0.001)	30	0.49(0.31-0.79, 0.003)	17	0.31(0.17-0.55, <0.001)
SES score 3-4	11544	107	0.60(0.44-0.82, 0.001)	74	0.67(0.46-0.98, 0.039)	46	0.47(0.31-0.73, <0.001)
SES score 0-2	4214	66	Reference	41	Reference	36	Reference

Note: SES: socioeconomic status; IBD: inflammatory bowel disease; UC: ulcerative colitis; CD: Crohn's disease. All HRs were adjusted for age, sex, ethnicity, study center, depression, anxiety and type 2 diabetes. The healthy lifestyle score consisted of never smoking, moderate alcohol drinking, healthy diet, regular physical activity, normal BMI, and healthy sleep duration. P for interaction was 0.084, 0.519 and 0.038 for IBD, UC and CD, respectively.

Table S12. Sensitivity analysis regarding risk of incident IBD, UC and CD associated with socioeconomic status by excluding IBD cases diagnosed within 2 years after baseline

Socioeconomic status	No. of events	No. of participants	HR (95%CI)
**Incident IBD**			
SES pattern			
High SES	281	76244	0.53(0.46-0.62)
Medium SES	1093	196973	0.79(0.72-0.87)
Low SES	707	100990	Reference

P for trend			<0.001
SES score, categorical			
5-6	691	153240	0.58(0.51-0.66)
3-4	1046	177486	0.75(0.66-0.85)
0-2	344	43481	Reference
P for trend			<0.001
SES score, continuous	2081	374207	0.85(0.83-0.88)
**Incident UC**			
SES pattern			
High SES	194	76244	0.54(0.45-0.64)
Medium SES	795	196973	0.84(0.75-0.94)
Low SES	491	100990	Reference
P for trend			<0.001
SES score, categorical			
5-6	488	153240	0.60(0.51-0.70)
3-4	754	177486	0.78(0.68-0.91)
0-2	238	43481	Reference
P for trend			<0.001
SES score, continuous	1480	374207	0.86(0.83-0.89)
**Incident CD**			
SES pattern			
High SES	99	76244	0.47(0.37-0.60)
Medium SES	363	196973	0.68(0.57-0.80)
Low SES	267	100990	Reference
P for trend			<0.001
SES score, categorical			
5-6	235	153240	0.49(0.39-0.61)
3-4	360	177486	0.65(0.53-0.80)
0-2	134	43481	Reference
P for trend			<0.001

SES score, continuous	729	374207	0.82(0.78-0.87)

Note: SES: socioeconomic status; IBD: inflammatory bowel disease; UC: ulcerative colitis; CD: Crohn's disease. For SES pattern, SES score (continous/categorical), all HRs were adjusted for age, sex, ethnicity, study center, depression, anxiety and type 2 diabetes. P for trend was calculated via 0,1 and 2 for low, medium and high SES pattern and median value of each category of SES score.

Table S13. Sensitivity analysis regarding risk of incident IBD, UC and CD associated with socioeconomic status by excluding IBD cases diagnosed within 3 years after baseline

**Socioeconomic status**	**No. of events**	**No. of participants**	**HR (95%CI)**
**Incident IBD**			
SES pattern			
High SES	262	76225	0.53(0.46-0.62)
Medium SES	1005	196885	0.78(0.71-0.87)
Low SES	656	100939	Reference
P for trend			<0.001
SES score, categorical			
5-6	638	153187	0.57(0.50-0.65)
3-4	965	177405	0.74(0.65-0.84)
0-2	320	43457	Reference
P for trend			<0.001
SES score, continuous	1923	374049	0.85(0.82-0.88)
**Incident UC**			
SES pattern			
High SES	178	76225	0.53(0.44-0.63)
Medium SES	729	196885	0.82(0.73-0.93)
Low SES	455	100939	Reference
P for trend			<0.001
SES score, categorical			
5-6	446	153187	0.59(0.50-0.69)

3-4	698	177405	0.79(0.67-0.92)
0-2	218	43457	Reference
P for trend			<0.001
SES score, continuous	1362	374049	0.85(0.82-0.89)
**Incident CD**			
SES pattern			
High SES	94	76225	0.48(0.37-0.61)
Medium SES	335	196885	0.67(0.56-0.79)
Low SES	248	100939	Reference
P for trend			<0.001
SES score, categorical			
5-6	219	153187	0.48(0.38-0.60)
3-4	331	177405	0.63(0.51-0.78)
0-2	127	43457	Reference
P for trend			<0.001
SES score, continuous	677	374049	0.82(0.78-0.87)

Note: SES: socioeconomic status; IBD: inflammatory bowel disease; UC: ulcerative colitis; CD: Crohn's disease. For SES pattern, SES score (continous/categorical), all HRs were adjusted for age, sex, ethnicity, study center, depression, anxiety and type 2 diabetes. P for trend was calculated via 0,1 and 2 for low, medium and high SES pattern and median value of each category of SES score.

Table S14. Sensitivity analysis regarding risk of incident IBD, UC and CD associated with socioeconomic status by excluding IBD cases diagnosed within 5 years after baseline

Socioeconomic status	No. of events	No. of participants	HR (95%CI)
**Incident IBD**			
SES pattern			
High SES	219	76182	0.53(0.45-0.63)
Medium SES	827	196707	0.78(0.70-0.87)
Low SES	534	100817	Reference

P for trend			<0.001
SES score, categorical			
5-6	525	153074	0.59(0.50-0.69)
3-4	805	177245	0.78(0.68-0.90)
0-2	250	43387	Reference
P for trend			<0.001
SES score, continuous	1580	374049	0.85(0.82-0.88)
**Incident UC**			
SES pattern			
High SES	147	76182	0.54(0.44-0.66)
Medium SES	599	196707	0.84(0.74-0.96)
Low SES	362	100817	Reference
P for trend			<0.001
SES score, categorical			
5-6	366	153074	0.62(0.51-0.75)
3-4	575	177245	0.84(0.70-0.99)
0-2	167	43387	Reference
P for trend			<0.001
SES score, continuous	1108	373706	0.86(0.82-0.90)
**Incident CD**			
SES pattern			
High SES	82	76182	0.49(0.38-0.64)
Medium SES	274	196707	0.65(0.54-0.79)
Low SES	203	100817	Reference
P for trend			<0.001
SES score, categorical			
5-6	181	153074	0.49(0.38-0.63)
3-4	278	177245	0.66(0.52-0.83)
0-2	100	43387	Reference
P for trend			<0.001

SES score, continuous	559	374049	0.82(0.77-0.87)

Note: SES: socioeconomic status; IBD: inflammatory bowel disease; UC: ulcerative colitis; CD: Crohn's disease. For SES pattern, SES score (continous/categorical), all HRs were adjusted for age, sex, ethnicity, study center, depression, anxiety and type 2 diabetes. P for trend was calculated via 0,1 and 2 for low, medium and high SES pattern and median value of each category of SES score.

Table S15. Sensitivity analysis regarding risk of incident IBD, UC and CD associated with socioeconomic status by additionally adjusting TDI quartiles

Socioeconomic status	No. of events	No. of participants	HR (95%CI)
**Incident IBD**			
SES pattern			
High SES	329	76292	0.59(0.51-0.68)
Medium SES	1250	197130	0.84(0.77-0.93)
Low SES	802	101085	Reference
P for trend			<0.001
SES score, categorical			
5-6	793	153342	0.63(0.55-0.71)
3-4	1196	177636	0.79(0.70-0.89)
0-2	392	43529	Reference
P for trend			<0.001
SES score, continuous	2381	374507	0.87(0.84-0.90)
**Incident UC**			
SES pattern			
High SES	225	76292	0.57(0.48-0.67)
Medium SES	914	197130	0.87(0.78-0.97)
Low SES	564	101085	Reference
P for trend			<0.001
SES score, categorical			
5-6	559	153342	0.61(0.53-0.71)
3-4	866	177636	0.80(0.69-0.91)

0-2	278	43529	Reference
P for trend			<0.001
SES score, continuous	1703	374507	0.87(0.84-0.90)
**Incident CD**			
SES pattern			
High SES	120	76292	0.57(0.45-0.71)
Medium SES	421	197130	0.76(0.65-0.89)
Low SES	302	101085	Reference
P for trend			<0.001
SES score, categorical			
5-6	276	153342	0.66(0.53-0.81)
3-4	419	177636	0.79(0.65-0.96)
0-2	148	43529	Reference
P for trend			<0.001
SES score, continuous	843	374507	0.86(0.82-0.91)

Note: SES: socioeconomic status; IBD: inflammatory bowel disease; UC: ulcerative colitis; CD: Crohn's disease. For SES pattern, SES score (continous/categorical), all HRs were adjusted for age, sex, ethnicity, study center, TDI quartiles, depression, anxiety and type 2 diabetes. P for trend was calculated via 0,1 and 2 for low, medium and high SES pattern and median value of each category of SES score.

Table S16. Sensitivity analysis regarding risk of incident IBD, UC and CD associated with socioeconomic status via competing risk model (No. of competing events= 28599, 28699, 28767 for IBD, UC and CD)

Socioeconomic status	No. of events	No. of participants	HR (95%CI)
**Incident IBD**			
SES pattern			
High SES	329	76292	0.57(0.50-0.65)
Medium SES	1250	197130	0.82(0.75-0.90)
Low SES	802	101085	Reference
P for trend			<0.001

SES score, categorical			
5-6	793	153342	0.61(0.53-0.69)
3-4	1196	177636	0.78(0.69-0.87)
0-2	392	43529	Reference
P for trend			<0.001
SES score, continuous	2381	374507	0.86(0.84-0.89)
**Incident UC**			
SES pattern			
High SES	225	76292	0.55(0.47-0.65)
Medium SES	914	197130	0.85(0.77-0.95)
Low SES	564	101085	Reference
P for trend			<0.001
SES score, categorical			
5-6	559	153342	0.60(0.52-0.70)
3-4	866	177636	0.79(0.69-0.91)
0-2	278	43529	Reference
P for trend			<0.001
SES score, continuous	1703	374507	0.86(0.83-0.89)
**Incident CD**			
SES pattern			
High SES	120	76292	0.53(0.42-0.66)
Medium SES	421	197130	0.72(0.62-0.84)
Low SES	302	101085	Reference
P for trend			<0.001
SES score, categorical			
5-6	276	153342	0.55(0.45-0.68)
3-4	419	177636	0.72(0.59-0.87)
0-2	148	43529	Reference
P for trend			<0.001

SES score, continuous	843	374507	0.85(0.80-0.89)

Note: SES: socioeconomic status; IBD: inflammatory bowel disease; UC: ulcerative colitis; CD: Crohn's disease. For SES pattern, SES score (continous/categorical), all HRs were adjusted for age, sex, ethnicity, study center, depression, anxiety and type 2 diabetes. P for trend was calculated via 0,1 and 2 for low, medium and high SES pattern and median value of each category of SES score.

Table S17. Sensitivity analysis regarding risk of incident IBD, UC and CD associated with socioeconomic status by excluding those who died during follow up

**Socioeconomic status**	**No. of events**	**No. of participants**	**HR (95%CI)**
**Incident IBD**			
SES pattern			
High SES	308	73720	0.57(0.50-0.66)
Medium SES	1134	185049	0.84(0.76-0.92)
Low SES	652	87824	Reference
P for trend			<0.001
SES score, categorical			
5-6	725	146345	0.64(0.56-0.74)
3-4	1076	163597	0.85(0.74-0.96)
0-2	293	36651	Reference
P for trend			<0.001
SES score, continuous	2094	346593	0.87(0.84-0.90)
**Incident UC**			
SES pattern			
High SES	211	73720	0.55(0.47-0.66)
Medium SES	836	185049	0.86(0.77-0.97)
Low SES	469	87824	Reference
P for trend			<0.001
SES score, categorical			
5-6	517	146345	0.63(0.54-0.74)

3-4	785	163597	0.85(0.73-0.99)
0-2	214	36651	Reference
P for trend			<0.001
SES score, continuous	1516	346593	0.87(0.83-0.90)
**Incident CD**			
SES pattern			
High SES	111	73720	0.54(0.43-0.69)
Medium SES	374	185049	0.74(0.63-0.88)
Low SES	237	87824	Reference
P for trend			<0.001
SES score, categorical			
5-6	247	146345	0.60(0.48-0.76)
3-4	370	163597	0.81(0.65-1.01)
0-2	105	36651	Reference
P for trend			<0.001
SES score, continuous	722	346593	0.86(0.81-0.90)

Note: SES: socioeconomic status; IBD: inflammatory bowel disease; UC: ulcerative colitis; CD: Crohn's disease. For SES pattern, SES score (continous/categorical), all HRs were adjusted for age, sex, ethnicity, study center, depression, anxiety and type 2 diabetes. P for trend was calculated via 0,1 and 2 for low, medium and high SES pattern and median value of each category of SES score.

Table S18. Sensitivity analysis regarding risk of incident IBD, UC and CD associated with socioeconomic status by excluding individuals with prevalent diabetes, depression and anxiety at baseline

**Socioeconomic status**	**No. of events**	**No. of participants**	**HR (95%CI)**
**Incident IBD**			
SES pattern			
High SES	298	69934	0.56(0.49-0.65)
Medium SES	1100	174783	0.82(0.75-0.91)
Low SES	627	82640	Reference

P for trend			<0.001
SES score, categorical			
5-6	706	138606	0.60(0.52-0.69)
3-4	1033	154856	0.79(0.69-0.90)
0-2	286	33895	Reference
P for trend			<0.001
SES score, continuous	2025	327357	0.86(0.83-0.89)
**Incident UC**			
SES pattern			
High SES	205	69934	0.55(0.46-0.66)
Medium SES	807	174783	0.85(0.76-0.96)
Low SES	448	82640	Reference
P for trend			<0.001
SES score, categorical			
5-6	500	138606	0.61(0.51-0.72)
3-4	757	154856	0.82(0.70-0.96)
0-2	203	33895	Reference
P for trend			<0.001
SES score, continuous	1460	327357	0.86(0.83-0.90)
**Incident CD**			
SES pattern			
High SES	106	69934	0.53(0.41-0.67)
Medium SES	367	174783	0.74(0.62-0.87)
Low SES	228	82640	Reference
P for trend			<0.001
SES score, categorical			
5-6	242	138606	0.53(0.42-0.66)
3-4	350	154856	0.68(0.55-0.85)
0-2	109	33895	Reference
P for trend			<0.001

SES score, continuous	701	327357	0.83(0.79-0.88)

Note: SES: socioeconomic status; IBD: inflammatory bowel disease; UC: ulcerative colitis; CD: Crohn's disease. For SES pattern, SES score (continous/categorical), all HRs were adjusted for age, sex, ethnicity, study center, depression, anxiety and type 2 diabetes. P for trend was calculated via 0,1 and 2 for low, medium and high SES pattern and median value of each category of SES score.

Table S19. Sensitivity analysis regarding risk of incident IBD, UC and CD associated with socioeconomic status by age-scaled Cox regression stratified by 5-years birth cohort

Socioeconomic status	No. of events	No. of participants	HR (95%CI)
**Incident IBD**			
SES pattern			
High SES	329	76292	0.56(0.49-0.64)
Medium SES	1250	197130	0.81(0.74-0.89)
Low SES	802	101085	Reference
P for trend			<0.001
SES score, categorical			
5-6	793	153342	0.59(0.52-0.67)
3-4	1196	177636	0.75(0.67-0.85)
0-2	392	43529	Reference
P for trend			<0.001
SES score, continuous	2381	374507	0.86(0.83-0.88)
**Incident UC**			
SES pattern			
High SES	225	76292	0.55(0.47-0.65)
Medium SES	914	197130	0.84(0.76-0.94)
Low SES	564	101085	Reference
P for trend			<0.001
SES score, categorical			
5-6	559	153342	0.58(0.50-0.68)

3-4	866	177636	0.77(0.67-0.88)
0-2	278	43529	Reference
P for trend			<0.001
SES score, continuous	1703	374507	0.86(0.83-0.89)
**Incident CD**			
SES pattern			
High SES	120	76292	0.51(0.41-0.63)
Medium SES	421	197130	0.70(0.60-0.81)
Low SES	302	101085	Reference
P for trend			<0.001
SES score, categorical			
5-6	276	153342	0.52(0.43-0.64)
3-4	419	177636	0.69(0.57-0.83)
0-2	148	43529	Reference
P for trend			<0.001
SES score, continuous	843	374507	0.84(0.80-0.88)

Note: SES: socioeconomic status; IBD: inflammatory bowel disease; UC: ulcerative colitis; CD: Crohn's disease. For SES pattern, SES score (continous/categorical), all HRs were adjusted for age, sex, ethnicity, study center, depression, anxiety and type 2 diabetes. P for trend was calculated via 0,1 and 2 for low, medium and high SES pattern and median value of each category of SES score.
